# Extracellular Lipids in the Lung and Their Role in Pulmonary Fibrosis

**DOI:** 10.3390/cells11071209

**Published:** 2022-04-03

**Authors:** Olivier Burgy, Sabrina Loriod, Guillaume Beltramo, Philippe Bonniaud

**Affiliations:** 1 INSERM UMR 1231 LNC, LabEX LipSTIC, Faculty of Medicine and Pharmacy, University of Bourgogne-Franche Comté, 21000 Dijon, France; olivier.burgy@u-bourgogne.fr (O.B.); sabrina_loriod@etu.u-bourgogne.fr (S.L.); guillaume.beltramo@chu-dijon.fr (G.B.); 2 Reference Center for Rare Lung Diseases, University Hospital Dijon-Bourgogne, 21000 Dijon, France; 3 Department of Pulmonary Medicine and Intensive Care Unit, University Hospital Dijon-Bourgogne, 21000 Dijon, France

**Keywords:** extracellular lipids, lipid metabolism, idiopathic pulmonary fibrosis

## Abstract

Lipids are major actors and regulators of physiological processes within the lung. Initial research has described their critical role in tissue homeostasis and in orchestrating cellular communication to allow respiration. Over the past decades, a growing body of research has also emphasized how lipids and their metabolism may be altered, contributing to the development and progression of chronic lung diseases such as pulmonary fibrosis. In this review, we first describe the current working model of the mechanisms of lung fibrogenesis before introducing lipids and their cellular metabolism. We then summarize the evidence of altered lipid homeostasis during pulmonary fibrosis, focusing on their extracellular forms. Finally, we highlight how lipid targeting may open avenues to develop therapeutic options for patients with lung fibrosis.

## 1. The Physiopathology of Pulmonary Fibrosis

Idiopathic pulmonary fibrosis (IPF) is a severe chronic and progressive disease of the lung parenchyma, causing significant morbidity through worsening dyspnoea, increasing cough, and overall irremediable functional decline [1]. The prognosis of IPF is poor, with a median survival time of less than five years. IPF development is classically viewed as an abnormal alveolar repair induced by an unknown triggering event leading to myofibroblast proliferation and extracellular matrix (ECM) accumulation in the lungs. Currently, only two drugs (pirfenidone and nintedanib) have been approved for the management of IPF. However, neither of these medications are able to reverse or even stop the progression of fibrosis, and so the patient’s lung function declines gradually over time [2,3]. The field is looking towards new avenues to develop therapeutics for patients with IPF.

The physiopathology of fibrosis is still not completely understood, although several factors and processes have been identified in the last few decades [4,5] (see [6] for a comprehensive review of the cellular and molecular mechanisms of IPF). The current working model is based on impaired cellular crosstalk among the different cells present in the lung [5]. Repeated damages to the lung epithelium activate an initial process of wound healing with the activation of epithelial cells, mainly alveolar type (AT)II cells. In this context, and under specific chronic conditions (e.g., aging, tobacco exposure, genetic predispositions), an aberrantly activated population of epithelial cells located in the distal lung emerges [7,8]. Those cells, also called aberrant basaloid cells, express specific keratin fibers (Krt8 and Krt15) and are believed to be stacked into a transitional state in which their healing activities are impaired and in which they transmit altered messages that activate neighboring cells such as fibroblasts [9]. Activated fibroblasts or myofibroblasts are mesenchymal cells with a crucial role in fibrosis since they produce extracellular matrix components, collagen being the main component. In addition, they are regarded as a population of cells with invasiveness and aggressive properties, with the expression of specific proteins such as the α-smooth muscle actin (α-SMA) or the hyaluronan synthases (HAS). Recent advances and single-cell transcriptomic approaches have highlighted the complexity of the fibroblast populations and their ability to promote repair or pathological extracellular matrix deposition [10,11]. The main source of (myo)fibroblasts is resident lung fibroblasts, which undergo activation to produce an altered extracellular matrix. Indeed, many other cell types seem to participate in the accumulation of activated (myo)fibroblasts, as observed in IPF [12]. ATII cells can experience genetic reprogramming, similar to the epithelial-to-mesenchymal transition (EMT) described in cancer, to acquire mesenchymal features and therefore contribute to the pool of activated fibroblasts [13]. Although many labs have described such activated ATII cells, the part of EMT-derived fibroblasts in IPF remains controversial [14]. Later, additional structural cells have been shown to differentiate into myofibroblast-like cells, such as pleural mesothelial cells. During IPF, pleural cells gain motility via the expression of the canonical myofibroblast marker α-SMA and can invade the lung parenchyma [15]. More recently, the plasticity of endothelial cells has been demonstrated. Under fibrotic conditions, endothelial cells in the lung can also undergo reprogramming and acquire mesenchymal properties via a process termed endothelial-to-mesenchymal transition [16]. A deeper understanding of this endothelial-to-mesenchymal reprogramming identifies and confirms potential therapeutic targets such as galectin-3 [17] Next to endothelium, vascular smooth muscle cells are also involved in pulmonary remodeling with the production of type I collagen [18,19]. In addition, a recent and growing body of literature has described the key role of immune cells in orchestrating the cellular interplay between epithelial cells and fibroblasts [20,21,22,23]. Under physiological conditions, immune cells and macrophages appear highly heterogenous with distinct populations, each of them involved in specific processes such as inflammation or matrix interactions [24]. Studies with single-cell RNA sequencing of lung tissue from patients with IPF confirmed the presence of immune cells and notably identified alterations in macrophages and the presence of plasma B cells in IPF [20,21,22,25,26]. Although the exact role of these cells during fibrosis is not fully understood, they are believed to contribute to the local pro-fibrotic milieu responsible for the abnormal activation of both epithelial and mesenchymal cells. In aging, the activation of alveolar macrophages can be driven by components of the microenvironment produced by epithelial cells [27]. The crosstalk between immune cells and structural (epithelial and mesenchymal) cells seems thus to be a double-edged sword.

The activation of these cells within the lung relies mainly on the interaction of these cells with abnormal ECM, which is a major hallmark in chronic pulmonary diseases since it serves as a source of cellular activator molecules [28]. These signals can lead to the (re)activation of developmental signaling during IPF, such as TGF-β1 or Wnt signaling pathways, which have an important role in fibrotic processes [4,29]. TGF-β1 is the canonical pro-fibrotic cytokine, responsible for fibroblast activation, structural cell reprogramming towards mesenchymal-like cells, and modulation of immune cell functions. In rodents, the adenovirus-mediated overexpression of TGF-β1 leads to a severe and progressive fibrotic response [30]. Consequently, approaches to counteract its signaling are an intense area of research [31]. Crosstalk between fibroblasts and epithelial cells in the lung appears to be key in maintaining tissue homeostasis, and this communication involves cytokines and extracellular mediators regulating developmental signaling. In physiological conditions, fibroblasts control the stemness of ATII cells via paracrine Wnt signaling [32]. The disruption of this crosstalk, for example, after a massive epithelial injury, leads to autocrine Wnt signaling in the ATII compartment and subsequent increased activation of these cells. In fibrosis, Wnt signaling is pathologically increased and can over-activate ATII cells. Beyond TGF-β1 and Wnt, other signaling are activated during IPF such as Sonic hedgehog, Notch, and the more recently unraveled Hippo YAP-TAZ pathway. YAP-TAZ are transcriptional cofactors activated by tissue stiffness and are overexpressed during IPF [33]. In fibroblasts, they promote invasiveness, production of ECM components, and subsequent fibrosis [33]. In epithelial cells, they contribute to the ATII to ATI differentiation [34]. Whether perturbation in YAP-TAZ signaling is involved in the inefficient ATII to ATI transition observed in IPF remains unclear.

The (re)activation of developmental signaling is currently seen as the consequence of many pathological phenomena which take place in the IPF lung. Insult of the pulmonary epithelium is believed as a key initiatory step of IPF. Why epithelium can be unable to repair itself is still largely unknown, although many mechanisms are linked to the disease [5]. Aging is an important factor in IPF and can give rise to non-functional and pro-inflammatory senescent cells [35]. Senescence has gained significant interest in the past decade, and strategies to clear senescent cells during IPF are actively tested [36,37,38,39]. Beyond senescence, hypoxia is a major hallmark of IPF, where scar tissue deposition hampers gas exchange. Hypoxia also gains attention as a predictive marker of the disease [40,41,42]. As a consequence, hypoxia leads to an increase in oxidative stress, dysfunction of mitochondria, and an unfolded protein response [43,44]. Altogether, these mechanisms impair the cell’s normal function and consequently cause organ failure.

The lung is an organ that is rich in lipids and active in terms of lipid metabolism. In the distal lung, a significant amount of lipids is found in the extracellular space, for example, forming the alveolar surfactant. Pulmonary surfactant is seminal in facilitating respiration by reducing surface tension during inspiration and preventing the collapse of alveoli. The surfactant is composed of up to 90% lipids, mainly produced by ATII cells and secreted towards the alveolar space [45,46]. Among the different lipids produced in the lung (see below), phospholipids, and particularly phosphatidylcholine (PC), are highly involved in the composition of surfactants [46]. In addition to phospholipids, fatty acids (FA) are important components of the pulmonary lipid content. Their significant heterogeneity (length, unsaturation) and their role as a precursor to producing active lipids explain their numerous roles within the cells. Arachidonic acid (AA) is a perfect example. Vehiculated thanks to many phospholipids, AA is an important precursor of active lipids such as prostaglandins, leukotrienes, or lipoxins. Many lipids are released in the extracellular milieu. This implies the presence of specific activation systems and the expression of lipid receptors and transporters such as ABCA, also involved in surfactant assembly within ATII cells [46]. Cholesterol represents around 10% of the lipid component of lung surfactants, and it has a crucial role in lowering surface tension [46]. Beyond pulmonary surfactants, lipids shape the extracellular milieu with which cells interact. The lung ECM is a highly complex patchwork of fibrous proteins (mainly collagens) and proteoglycans (heparin, hyaluronic acid) as well as other glycosylated proteins such as fibronectin, laminin, and osteopontin (OPN). There are many examples of the intimal link between lipids and ECM. In the liver, OPN, which accumulates in fibrotic ECM, regulates the metabolism of PC and cholesterol by acting on hepatic P450 cytochrome expression [47]. Mice deficient for *Opn* are less prone to develop fibrosis and have decreased PC content [47]. In the lung, *OPN* is up-regulated in PASMCs stimulated with S1P [48]. The up-regulation of *OPN* is crucial in S1P-induced PASMCs proliferation. The activation of PPARγ suppresses the effects of S1P on OPN expression in PASMCs. Several master regulators, such as Lipin-1/SREBP, have been identified in line with these studies showing the regulation of lipid metabolism by ECM [49]. This regulatory crosstalk seems to work both ways as active lipids also drive the production of ECM components. For example, lysophosphate acid, a bioactive lipid, regulates the production of ECM production (collagen, fibronectin) via the transcriptomic activation of Yes-associated proteins (YAP)/PDZ-binding domain (TAC) in trabecular meshwork cells [50].

In pathological conditions, lipid production and metabolism are dysregulated. Furthermore, a growing body of literature identifies the alteration of lipids and their metabolic pathways as a central feature in lung fibrosis [51,52]. During wound healing and organ fibrosis, lipids serve as mediators in various processes such as the activation or resolution of inflammation. Moreover, these lipids can mediate both paracrine and endocrine cell–cell communication.

In this review, we will describe the various lipids present in the lung and explain their metabolic pathways. Then, we will discuss the abnormalities found in lipid composition and metabolism during various pathophysiological processes related to fibrogenesis before addressing how lipids may open therapeutic avenues for lung fibrogenesis and IPF.

## 2. Overview of Pulmonary Lipids and Their Metabolism

Lipids are a heterogenous group of hydrophobic molecules that can be classified into four main classes: (1) glycerides, including phospholipids, (2) fatty acids (FA), (3) non-glyceride lipids including cholesterol and sphingolipids, and (4) complex lipids (lipoproteins).

### 2.1. Phospholipids

Phospholipids constitute a class of amphiphilic molecules composed of a central glycerol cytoskeleton branched with two fatty acids (sn-1 and sn-2) and a polar group termed as “the head”. This group can be choline, serine, ethanolamine, or inositol. As stated above, pulmonary surfactant is essentially composed of lipids and mainly PCs such as the dipalmitoyl-PC [45]. The synthesis of PCs relies on choline, which is trimethylamine with many functions in cell proliferation, differentiation, migration, and apoptosis [53,54,55]. Increased choline uptake is observed in cancer cells in various organs, including lung carcinoma cells or liver cirrhosis [56,57,58]. Several pathways leading to the synthesis of PCs have been described, with the de novo synthesis pathway known as the Kennedy or cytidine-diphosphate (CDP)-choline pathway [59]. Within the lung, choline is imported from the blood towards ATII cells [60,61,62,63]. Intracellular choline first undergoes phosphorylation before transfer to a cytidylyl triphosphate (CTP) to obtain CDP-choline, which in turn is branched into diacylglycerol (DAG) to produce PC. Next to their role as structural lipids, phospholipids, including PCs, also serve as a source of secondary messengers and active mediators, mainly via FA transportation.

### 2.2. Fatty Acids

FAs are classified as saturated or unsaturated and also by the length of the carbon chain, including short-chain FAs (≤6 carbons), medium-chain FAs (6–12 carbons), long-chain FAs (12–22 carbons), and very-long-chain FAs (more than 22 carbons) [64]. Some FA can be generated endogenously from acetyl-coA and acetyl-coA carboxylase (ACACA) to produce malonyl-coA, which is the active donor for FA biosynthesis, which is elongated to generate a carbon chain [65,66]. The first product is released into the cytoplasm as a 4-carbon carboxylic acid (butyric acid, C4:0). Repeated elongation cycles of the butyric acid molecule allow the production of long carbon chains up to palmitic acid (C16:0) or stearic acid (C18:0). ELOVL enzymes are responsible for the elongation of FAs and are classified into seven groups based on their substrates [67,68]. Saturated short carbon chain FAs (e.g., palmitic acid) are substrates for ELOVL6. In parallel, saturated long and very long carbon chain FAs (e.g., stearic acid) are substrates for ELOVL1 [68,69], while ELOVL3 and ELOVL7 elongate saturated and unsaturated long carbon chains. ELOVL2 and five elongated unsaturated carbon chains such as arachidonic acid (C20:4, AA) [70]. 

FA can be released from phospholipids via the Lands’ cycle, where phospholipids are converted into lysophospholipids (reaction accompanied by the release of the sn-2 FA) under the action of the A2 phospholipase [58,71]. Lysophospholipids can, in turn, be used by lysophosphatidylcholine acyltransferase (LPCAT) enzymes to branch another FA in the sn-2 position and then form another complete phospholipid, or they can be processed to generate active extracellular lipids. Autotaxin is a secreted enzyme with lysophospholipase D (LPD) activity. It hydrolyzes extracellular lysophospholipids such as lysophosphatidylcholine (LPC) to obtain lysophosphate acid (LPA), a known active lipid mediator [72,73,74]. Indeed, extracellular LPA will bind and activate G protein-coupled transmembrane receptors. Six receptors, LPA1 to LPA6, have been described so far [72]. Although circulating LPA levels correlate with autotaxin concentrations, LPA can be generated after hydrolysis of phosphatidic acid (PA) by phospholipases A1 and A2. This reaction releases the FA in the sn-1 and sn-2 positions of the PA to produce LPA [51,72].

Among FAs, arachidonic acid (AA) is a major inflammatory mediator seeing as it can serve as a precursor to the generation of eicosanoids, including prostaglandin, leukotriene, and lipoxins [75,76]. These active lipids control tissue inflammation. The bioavailability of AA is major, and AA is the main FA located in the sn-2 position of phospholipids found linked to the membranes [49]. Within cells, hormonal, physical, or chemical stimuli can activate phospholipase A2, which is responsible for the hydrolysis of the sn-2 FA on phospholipids, leading to the release of AA. Then, free AA is metabolized by prostaglandin synthase isoforms COX-1 and COX-2. COX-1 is the main isoform expressed constitutively in cells, while the isoform COX-2 is activated during inflammation. COX enzymes produce prostaglandin H2 (PGH2), which serve as a precursor to producing other prostaglandins [77]. Alternatively, AA can also be converted into 5-hydroperoxyeicosatetraenoic (HPETE) acid by lipoxygenase, mainly the 5-lipoxygenase (5-LOX, also known as ALOX) to produce leukotrienes [78]. The lipoxins are pro-resolving lipids that play a vital role in reducing excessive tissue injury and chronic inflammation [79]. Lipoxins are synthesized by two pathways from AA and involve different lipoxygenases (5-, 15- or 12- LOX). The first pathway is the conversion of leukotriene A4 into lipoxin A4 and B4 by 12-LOX. In the second pathway, lipoxin can be obtained by the conversion of AA into 15-HPETE by 15-LOX, successively by the conversion of 15-HPETE into 15-OH-leukotriene A4 to produce lipoxin A4 and B4. 

The extracellular localization of lipids implies a system of recognition through specific surface receptors. ABCA3 is a lipid transporter with an ATP binding cassette believed to play a key role in the homeostasis of pulmonary surfactants [80]. CD36 is a transmembrane glycoprotein also known as FA translocase or scavenger receptor class B2 [81]. CD36 is localized in cellular lipid rafts. The FA moves through the bilayer membrane from the outer to the inner leaflet by a flip-flop process in order to be metabolized. CD36 facilitates the transport of long carbon chain FAs such as docosahexaenoic acid (DHA, C22:6) and eicosapentaenoic acid (EPA, C20:5) through the plasma membrane [49]. CD36 is expressed in various cells, including breast and eye epithelial cells, endothelial cells, enterocytes, insulin-responsive cells, and hematopoietic cells such as platelets, monocytes, and macrophages [81,82,83]. The expression of CD36 is associated with the regulation of lipid metabolism and innate immunity. CD36 is involved in tissue inflammation, intestinal fat absorption, lipid storage in adipocytes, and diseases such as obesity, Alzheimer’s, or diabetes [84,85]. 

### 2.3. Non-Glyceride Lipids and Lipoproteins

Ceramides are essential constituents of the plasma membrane. They regulate cell signaling, proliferation, differentiation, and apoptosis [86,87]. Ceramides belong to the sphingolipid family, which is based on sphingosine (fatty alcohol containing an ethylenic bond and an 18-carbon chain) linked to a long-chain FA [88,89]. Ceramides can be produced in the endoplasmic reticulum from a palmitoyl CoA molecule or be recycled in the lysosome via the hydrolysis of glycosphingolipids or sphingomyelin by sphingomyelinase. In parallel to ceramides, sphingosine-1-P (S1P) has gained increasing attention. S1P is produced after ceramidases produce sphingosine from ceramides [90]. Sphingosine is then used by sphingosine kinases (SphK1, SphK2) to form S1P. S1P is a class of bioactive lipids acting both as intracellular and extracellular mediators. S1P regulates several cellular processes involved in cell cycles, apoptosis as well as invasion, migration, and resistance to cancer therapy [91,92,93]. S1P can also be used as a biomarker of diseases such as Alzheimer’s [94]. 

Beyond phospholipids, fatty acids, and their derivatives, cholesterol is a major part of the lipid composition of mammals [95]. Cholesterol is an important lipid involved in the regulation of membrane fluidity and serves as a precursor for steroid hormones synthesis. This steroid can be obtained through diet or synthesized de novo in the intestine and mostly in the liver. 3-Hydroxy-3-methylglutaryl(HMG)-CoA reductase has been identified as a key enzyme in de novo cholesterol synthesis [96]. The activity of this enzyme can be inhibited by a class of pharmacological inhibitors called statins. As cholesterol is a seminal compound of membranes throughout the body, a complex system regulates its transport from the liver to the peripheral tissue and back [97]. In brief, low-density lipoproteins (LDL) are loaded in the liver with cholesterol and other lipids (FA) that are mainly transported as triglycerides and phospholipids. LDL then distributes lipids to the peripheral tissue. In parallel to LDL, high-density lipoproteins (HDL) take up lipids, mainly cholesterol, from the peripheral tissue and transport them back to the liver for recycling or elimination. This cycle appears to be highly regulated, and its dysfunction results in atherosclerosis and systemic inflammation [97].

## 3. Dysregulated Lipids and Their Metabolism during Lung Fibrosis

**Figure 1:**
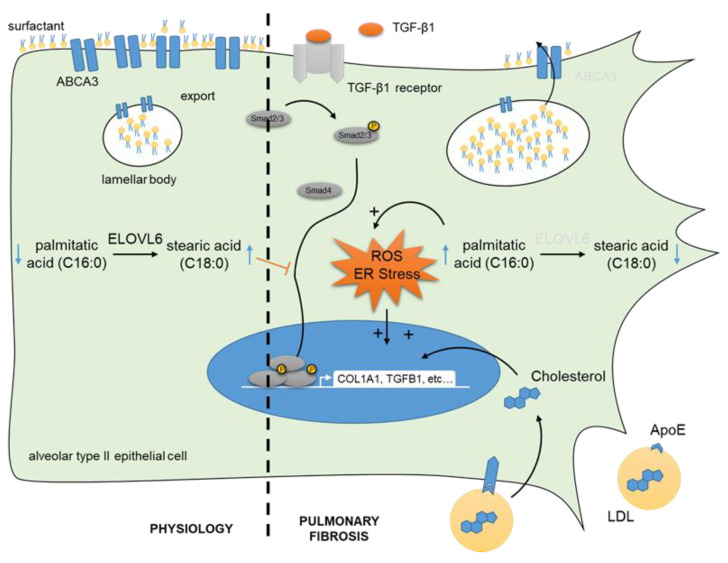
**Altered lipid metabolism during fibrosis contributes to the activation of alveolar epithelial cells.** Under physiological conditions, the elongation enzyme ELOVL6 allows fatty acid elongation and therefore promotes high intracellular stearic acid vs. palmitic acid. Stearic acid interferes with pro-fibrotic TGF-β/Smad signaling (red inhibition arrow). In parallel, ATII cells produce surfactant lipids (mainly PCs) which will be stocked in lamellar bodies and exported in the extracellular milieu in an ABCA3-dependant mechanism. During pulmonary fibrosis, ELOVL6 expression is decreased, favoring the accumulation of palmitic acid. High intracellular palmitic acid induces oxidative and ER stress, thus promoting TGF-β/Smad signaling and cell activation. This is enhanced by the accumulation of cholesterol and its derivates during fibrosis, which activates the expression of collagen and other ECM components. In addition, surfactant lipids accumulate within the cell due to decreased expression of ABCA3 under fibrotic conditions. This also leads to impaired surfactant formation in pulmonary fibrosis.

In recent years, altered lipid metabolism has been confirmed in pulmonary fibrosis as well as in other lung-related conditions, including ARDS [45,98]. Evidence of dysregulated expression of proteins linked to lipid metabolism during lung fibrosis have been compiled in Table 1. Unbiased transcriptomic approaches in tissue from patients with IPF identified key alterations in genes involved in lipid metabolism and regulation [20,22,45]. Using single-cell RNA sequencing on pulmonary cells derived from patients with IPF, an enrichment of gene ontology terms linked to lipids has been shown in tissue-resident alveolar macrophages [20]. As mentioned above, ABCA3 is a seminal transporter involved in lipid secretion and thus surfactant homeostasis (Figure 1). In ATII cells, the expression of genes involved in lipid transport such as ABCA3 is decreased during IPF [99]. Among genetic interstitial lung diseases (ILDs), more than 200 mutations have already been described in *ABCA3*, located on chromosome 16. Patients present heterogeneous phenotypes, from lethal neonatal respiratory distress syndrome to childhood and rarely adult interstitial lung disease [100]. The same observation regarding Abca3 has been made in mice subjected to bleomycin, who also displayed decreased intracellular cholesterol, free fatty acids, and triglycerides [101]. Consistent with the link between senescent ATII and IPF, single-cell RNA sequencing approaches in mice also revealed a disturbed lipid regulation by SREBPs in aged ATII, together with increased cholesterol synthesis [102]. In fibroblasts, the deregulation of lipid metabolism leads to the accumulation of pathological myofibroblasts [103]. 

Beyond transcriptional studies focusing on the expression of metabolic pathways linked to lipids, a growing body of literature has investigated the alteration of the lipids themselves during fibrosis. It appears that phospholipid classes have a unique distribution between the lung cells, with ATII mainly expressing PG and PC, alveolar macrophages expressing PE, and bronchial epithelial cells expressing PI [110]. The comprehensive profiling of the lung tissue collected on mice subjected to bleomycin highlighted significant differences [111]. In comparison to the control tissue, the fibrotic samples were mainly enriched in PC, PG, and cholesterol ester at D7 post-injury, with PC increasing from D7 to D21. Such lipid quantification was also applied in the bleomycin model together with interventional approaches. In a paper investigating VEGF inhibitors as potent anti-fibrotic in the bleomycin model, analysis of the mouse lung revealed increased lipids, such as PC linked to long fatty acids (e.g., PC 36:4) following bleomycin exposure, which returned to normal upon treatment with a VEGF inhibitor [112]. These variations were confirmed by the analysis of genes and proteins involved in the metabolism of these specific lipid species. 

In addition to unbiased lipidomics, several studies have highlighted the accumulation of specific FAs, such as palmitic acid, during IPF [113]. FA modification is a central event in lipid biogenesis. One possible modification is elongation, which determines lipid function and metabolic activity. The role of ELOVL6 in pulmonary fibrosis has been of particular interest because this enzyme is responsible for the conversion of palmitate (C16:0) into stearate (C18:0). ELOVL6 is significantly downregulated in the lungs of patients with IPF and in mice challenged with bleomycin [113]. In addition, stearic acid inhibits the activation of fibroblasts with reduced TGF-β1-activated Smad signaling as well as ECM and collagen production [114]. In parallel, Elovl6-deficient mice exhibit an altered FA composition in the lung accompanied by a more severe fibrotic response upon bleomycin exposure compared with wild-type littermates. In alveolar epithelial cells, the accumulation of palmitic acid results in oxidative stress, subsequent TGF-β1 production, and apoptosis (Figure 1). Consistently, it has been reported that a palmitic acid-enriched high-fat diet increases mortality in mice subjected to bleomycin [113]. In this model, the increased stress of the endoplasmic reticulum in alveolar epithelial cells has been observed, which was dependent on the presence of the lipid receptor CD36. Nevertheless, the association between increased levels of palmitic acid following a high-fat diet and increased mortality after bleomycin remains not completely understood since fatty diets are also linked to systemic inflammation.

Cellular stress is a common phenomenon observed during IPF, mainly in aberrantly activated ATII cells [5]. Interestingly, stress such as ER or mitochondrial stress is also linked to lipid metabolism disorders. Impaired mitochondria is a major hallmark of aberrantly activated ATII during IPF. Mice harboring deletions for mitofusin proteins, orchestrating mitochondria fusion and homeostasis, exhibit increased fibrosis after bleomycin exposure compared with wild-type mice [115]. Mechanistically, mitofusin inhibition hampers phospholipids, particularly PSs in ATII cells, showing the intimate relationship between cellular stress and lipid metabolism. In ATII cells, the stress of the endoplasmic reticulum that is induced during fibrosis promotes lipid production that is dependent on the stearoyl-coenzyme A desaturase 1 enzyme [116]. In those cells, lipid production is necessary to resolve endoplasmic reticulum stress. The pharmacological inhibition of this desaturase exacerbates ER stress in epithelial cells and then potentializes fibrosis.

Cholesterol and related vesicular transport systems are major bioactive lipid species that are dysregulated in many chronic lung diseases, including COPD or asthma [117,118]. The link between cholesterol and fibrosis is becoming progressively clearer. The development of high-fat diet-induced hypercholesterolemia in ApoE null mice leads to systemic inflammation and further lipid accumulation within the lung, causing subsequent fibrosis [119]. In vitro, the cholesterol derivatives 27- or 25-hydroxycholesterol induce α-SMA and type I collagen expression in mesenchymal cells (Figure 1) [117,118]. In both studies, blockade of the TGF-β signaling abolishes the activation properties of these derivatives. In alveolar epithelial cells, the addition of HDL enhances proliferation and migration properties with the activation of AKT and ERK signaling pathways [120]. It should be noted that HDL is a well-known system for the transport of cholesterol from the peripheral tissues back to the liver.

## 4. Extracellular Lipids as Important Regulators of Fibrosis Progression 

**Figure 2:**
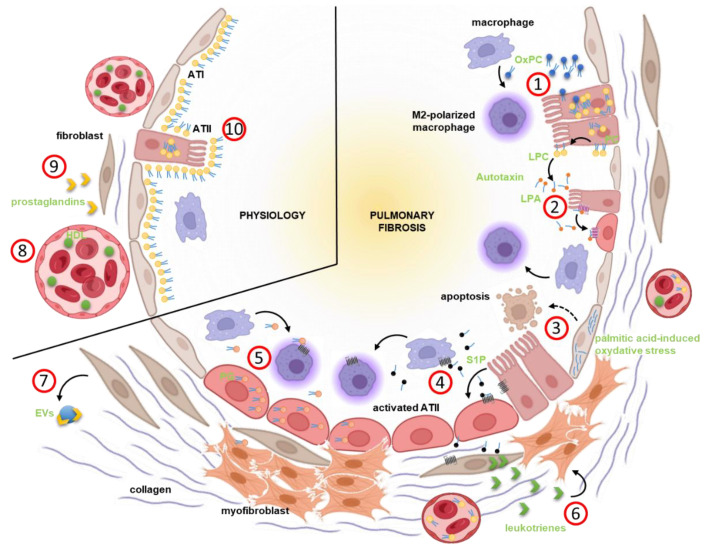
**The role of extracellular lipids in the pathogenesis of pulmonary fibrosis.** During pulmonary fibrosis, (**1**) impaired secretion of the surfactant lipids, mainly phosphatydil-choline (PC) promotes the accumulation of oxidized phospholipids, mainly oxidized-PC (OxPC). (**2**) PC are also a source of lysophosphate acid (LPA) after processing by autotaxin, which is increased during fibrosis. (**3**) The accumulation of palmitic acid in alveolar epithelial cells results in major oxidative stress and ultimately apoptosis. (**4**) Sphingosine-1-phosphate (S1P) accumulates in the extracellular space and promotes the EMT of ATII cells. Further, (**5**) activated ATII cells produce large amount of phosphatydil-glycerol. All these lipids contribute to the polarization of local macrophages towards a profibrotic M2-phenotype or the activation of ATII cells. In addition, S1P has a supplemental role in the activation of aberrant ATII cells. Altogether, cell activation turns fibroblasts into pathological (myo)fibroblasts which (**6**) produce high levels of arachidonic acid derivatives with a high leukotriene to prostaglandin ratio. Of note, (**7**) inflammation-primed fibroblasts also secrete extracellular vesicles carrying prostaglandins. The increase in pulmonary lipids during fibrosis is linked to increased circulating PC. In normal conditions, (**8**) lower levels of blood PC are likely to be found with increased circulating HDL, and (**9**) arachidonic acid metabolism in fibroblasts results in leukotriene overproduction. (**10**) ATII cells produce surfactant-forming lipids (mainly PCs) to participate in lung surfactant homeostasis. Cell types (black) and lipids (light green) are labeled.

Apart from its crucial role in regulating the surface tension of the alveolar walls, surfactant lipids also manage the interplay between ATII cells and the local immune system. For example, specific PC and P-Glycerol entities are capable of modulating alveolar macrophage polarization and function (Figure 2) [121,122]. Surfactant homeostasis and lipid composition are often disturbed in pulmonary diseases [123].

Lipid profiling applied to bronchoalveolar lavage fluid (BALF) in experimental rodent models has uncovered significant alterations and the accumulation of lipids in the extracellular space within the lung [111]. These lipids include a large range of classes, from phospholipids to eicosanoids. In parallel, the role of oxidized lipids has emerged in many chronic pulmonary diseases such as asthma or lung fibrosis [101,124]. Upon bleomycin exposure, secreted oxidized PC can be measured in the BALF of mice [101]. Consequently, lipid-loaded foam cells accumulate in the lungs of those mice, mainly in proximity to ATII cells. The presence of these oxidized lipids seems to be crucial for fibrosis development, seeing as oxidized PCs mitigated macrophage polarization toward an M2-phenotype (Figure 2). Intra-tracheal instillation of oxidized PCs in mice induces a severe fibrotic response [101]. These data suggest that during pulmonary fibrosis (aberrantly activated), ATII cells secrete altered and oxidized lipids into the lung, which drive a pro-fibrotic, M2-type reprogramming of local macrophages, contributing to fibrogenesis. This highlights the role of lipids in shaping the genetic program of the cells to further promote fibrosis. This epithelial-macrophage crosstalk remains to be described, and the potential role of fibroblasts in this impaired communication warrants further investigation.

Lysophosphatidic acid (LPA) is a major bioactive phospholipid acting via G protein-coupled surface receptors, and it is involved in many illnesses, including cancer and fibrosis [52,125,126]. LPA is increased in wild-type mice subjected to bleomycin compared with non-treated mice [127]. LPA can be generated by autotaxins, which are secreted hydrolases converting LPC into LPA (Figure 2) (see chapter 2, [128]). Autotaxin is overexpressed in human IPF as well as after bleomycin exposure in rodents [105]. Autotaxin knock-out in bronchial epithelial cells or macrophages consistently decreased collagen accumulation in mice subjected to bleomycin. Mice lacking LPA receptor 1 exhibit a rapidly observable default in alveolarization [129]. In alveolar epithelial MLE12 cells, the stabilization of the LPA receptor 1 increases cell migration and ERK signaling (Figure 2) [130]. Furthermore, the administration of an LPA antagonist consistently diminishes bleomycin-induced fibrosis by interfering with the activation of myofibroblasts [127]. LPA1 inhibitors are currently being tested in patients with IPF (see chapter 6).

As mentioned above, arachidonic acid is a master fatty acid thanks to its ability to serve as a precursor for complex lipid mediators such as prostaglandins and leukotrienes. Those so-called eicosanoids seem to be mainly produced by immune cells and appear to be important factors in structural cell activation and thus fibrosis development [52,131]. The production of these mediators seems to be dysregulated during lung fibrosis, mainly resulting in enhanced synthesis of leukotrienes versus prostaglandin E(2), establishing a pro-fibrotic environment (Figure 2) [78]. Whether dysregulated eicosanoid leads to fibrosis development or the other way around seems still unclear. Tissue stiffness, a major hallmark of fibrotic tissues, down-regulates COX-2 expression and therefore diminishes prostaglandin production [108]. In parallel, Cox-2 repression appears to be controlled by a number of mechanisms, including epigenetic and MAP3K8 signaling [132,133].

The role of leukotrienes as contributors to the development of fibrosis has been well described. Mice overexpressing leukotriene C4 synthase had worsened pulmonary fibrosis after bleomycin exposure compared with wild-type mice [134]. Inversely, leukotriene signaling blockade using antagonist of the leukotriene receptor or genetic deletion of the enzyme involved in their production (such as 5-lipoxygenase) attenuates bleomycin-induced fibrosis [135,136,137]. Further, the inhibition of leukotriene production impairs Smad-dependent TGF-β signaling in fibroblasts [138]. Leukotrienes are pro-inflammatory molecules that are part of the altered secretome of senescent cells observed during IPF [36,37]. In IPF, 50% of cells expressing the principal enzyme of the leukotriene synthesis ALOX5 also express the senescence marker p16 [106]. Conditioned media from radiation-induced senescent fibroblasts triggers fibrosis pathways in fibroblasts, and this was abolished upon ALOX5 inhibition [106]. Interestingly, senescent fibroblasts isolated from the lungs of patients with IPF aberrantly produce leukotrienes and no prostaglandins (Figure 2). This disbalance is believed to be one of the mechanisms of fibrosis.

Mice with a genetic deletion of the prostaglandin synthase in hematopoietic cells have increased fibrosis after bleomycin compared with normal mice [139]. This is in line with the protective role of prostaglandins in pulmonary fibrosis, which is not completely understood. In the bleomycin model, prostaglandin E2 supplementation starting at D14 post bleomycin did not impact fibrosis development nor increase fibrosis resolution in the model [140]. However, mice that received prostaglandin E2 before the bleomycin challenge developed less severe fibrosis, while mice lacking the prostaglandin E2 synthase had fibrosis similar to their wild-type littermates [140,141]. This surprising result suggests that prostaglandin may be beneficial during the model development and does not interfere with the mechanisms involved in disease progression. This is consistent with the regulation of prostaglandin production by alveolar epithelial cells. Indeed, extracellular ATP can trigger either prostaglandin or pro-inflammatory IL-6 secretion in those cells depending on the activation or not of ionotropic P2X receptors [142]. Many studies support the idea that prostaglandins have a role in fibroblast biology. Prostaglandins reduce the activation of fibroblasts in myofibroblasts by decreasing their proliferation and inhibiting the production of ECM components [143,144,145,146]. However, the biology behind prostaglandin production and action remains to be fully understood. For instance, the transfer of prostaglandin E2 from epithelial cells or T-lymphocytes co-cultured together with fibroblasts diminishes TGF-β1-triggered activation of the mesenchymal cells [147,148]. Altogether, these studies identify prostaglandins as seminal mediators in inter-cellular crosstalk, showing how alveolar epithelial cells and immune cells direct fibroblast activation. ILD fibroblasts can be resistant to prostaglandin E2 [107]. In these cells, resistance to prostaglandins is explained by the hypermethylation of the prostanoid E2 receptor, which can be restored using a DNA methylation inhibitor [149]. Interestingly, patients with prostaglandin-resistant fibroblasts are also patients with more altered lung function [107]. In parallel, IPF fibroblasts also have nonfunctional COX-2 and therefore lack the ability to produce prostaglandins. Impaired prostaglandin responsiveness also affects the protective role of the plasminogen activation [150,151].

In addition to eicosanoids, other circulating lipids such as S1P have emerged as important players in fibrosis development and progression in the lung and other organs [52,90,152,153,154]. In patients with IPF, S1P is increased in the BALF or in the blood compared with control patients or healthy subjects (Figure 2) [155]. The same observations have been made in rodents exposed to bleomycin [156]. More importantly, the accumulation of S1P in BALF correlates with lung function parameters in IPF. Mechanistically, S1P enhances the reprogramming (EMT) of ATII cells induced by TGF-β1 (Figure 2). This underlines that aberrantly activated ATII cells secrete extracellular bioactive lipids able to enhance their trans-differentiation (EMT). Mice with a genetic deletion of the acid sphingomyelinase, an enzyme required to produce ceramide and thus S1P, have reduced pulmonary collagen after bleomycin compared to control mice [156]. Likewise, the S1P lyase, an enzyme able to degrade S1P, is overexpressed in fibrotic tissues during IPF or after bleomycin in mice [109,157]. The genetic deletion of S1P lyase in mice promotes fibrosis while its overexpression in vitro counteracts TGF-β1-induced cell activation and activates autophagy in fibroblasts. Consistently, the reduced expression of S1PL in PBMCs from patients with IPF correlates with the severity of the disease in those patients [109]. S1P can signal by binding to five surface G protein-coupled receptors, and many studies have investigated what occurs when there is a deficiency of these receptors. The adenovirus-mediated inhibition of S1P receptor 3 in mouse lungs leads to reduced inflammatory cell infiltration, histology change, and collagen accumulation in a model of radiation-induced pulmonary fibrosis [158]. The expression of S1P receptor 3 is regulated by the microRNA-495-3p. In alveolar epithelial cells, the microRNA-495-3p mimic decreases S1P receptor 3 and hampers the activation of those cells [158]. In mice, the genetic deletion of the enzyme involved in S1P generation, sphingosine kinase (SPHK)1, in fibroblasts or alveolar epithelial cells, reduces bleomycin-induced fibrosis [159]. The pharmacological inhibition of SPHK1 reduces fibrosis in vivo. Mechanistically, SPHK1 inhibition counteracts the activation of YAP signaling that is triggered by TGF-β1 or bleomycin exposure in fibroblasts or epithelial cells in vitro [159]. In this study, disturbing SPHK1 expression in endothelial cells had no effect. In parallel, invalidation of the S1P receptor 1 gene in endothelial cells resulted in worsened fibrosis after bleomycin exposure [160]. Compared to controls, S1pr1-/- mice have increased vascular permeability and immune cell influx within the lung as well as coagulation activation. Interestingly, increased S1P/S1PR1 axis in ApoM overexpressing transgenic mice did not reduce experimental fibrosis compared with normal mice [160]. S1pr2 null mice exhibited less inflammation and fibrosis upon bleomycin compared with wild-type mice [161]. The pharmacological inhibition of the S1P receptor 2, using JTE-013, diminishes the activation of alveolar epithelial cells induced in vitro by TGF-β1 [161]. However, the use of a genetically engineered mouse model to track S1pr2 expressing cells shows that this receptor is not limited to structural cells. Alveolar macrophages also express this S1P receptor 2 during fibrosis [162]. The analysis of BALF cells from S1pr2-/- mice subjected to bleomycin showed increased IL-13 signaling [162]. This finding is consistent with the hypothesis that S1P receptor 2 engagement on macrophages promotes fibrosis by polarizing those cells towards a pro-fibrotic M2 phenotype (Figure 2) [162,163].

Cellular communication is key in the onset and development of IPF. Several mechanisms of cell-to-cell crosstalk have been described in the disease, one of them being extracellular vesicles (EVs) [5]. EVs, including exosomes, are membranous vesicles secreted by all cells and act as transporters for molecules (also called cargoes) such as proteins, nucleic acids, or lipids [163]. A growing body of literature shows that EVs are a key component in the pathobiology of IPF. Those vesicles accumulate in the lungs of patients with IPF [164], and they harbor specific cargoes of proteins and microRNA [164,165,166,167,168,169]. Mechanistically, fibrosis-derived EVs activate the mechanism of fibrosis, such as fibroblast proliferation and activation, by controlling developmental signaling such as Wnt or TGF-β1 [164,167]. This seems to be highly dependent on the crosstalk driven by these vesicles. Macrophage-derived EVs appear to have anti-fibrotic properties through the transfer of specific microRNA to epithelial cells [170]. Vesicles secreted by bronchial epithelial cells also transfer microRNA and hamper fibroblast activation and senescence [171]. The characterization of these vesicles, the crosstalk they mediate, and the understanding of how cargos are packed into them could potentially lead to the identification of new therapeutic targets in IPF. EVs carry different molecules, such as lipids, that participate in disease mechanisms. These vesicles accumulate in the BALF of patients with asthma, and significant lipidomic changes, mainly on ceramides and PG, have been observed in EVs from asthmatics compared with healthy controls [172]. EVs isolated from asthmatics transport not only specific lipids but also trigger leukotriene production in bronchial epithelial cells, which demonstrates the close link between EVs and lipid dysregulation during chronic lung diseases [173]. In IPF, EVs also carry a distinct cargo of microRNA and proteins [165,174]. The analysis of the proteome of the IPF-EVs shows the presence of proteins involved in lipid metabolism [165]. Furthermore, prostaglandins are found in EVs from fibroblasts activated with IL-1β (Figure 2) [175]. This finding is consistent with the hypothesis of paracrine signaling in which inflammation-primed fibroblasts secrete EVs to communicate with and limit the activation of the surrounding mesenchymal cells.

## 5. Lipids and Pulmonary Fibrosis, The Hope for Potential Biomarkers

Patient heterogeneity and stratification remain a challenge in IPF. Over the last few years, there has been an emphasis on this aspect in order to better classify patients. More particularly, studies have investigated whether lipid profiling can be used as biomarkers or prognostic tools in pulmonary fibrosis. Lipid transport has been identified as a key component of the proteins identified in the BALF of a subset of patients with pulmonary fibrosis [176]. At the circulating level, the quantification of HDL particles correlates inversely with IPF severity and prognosis (Figure 2) [177]. This supports data obtained from larger cohorts such as in the MESA study, where high levels of HDL were associated with lower fibrosis biomarkers (e.g., MMP7, SP-A) and fibrosis assessed by CT [178]. The mechanism underlying this association remains to be fully elucidated, but it is known that lipids obtained through diet and transported by circulating lipoproteins can influence pulmonary surfactant composition [179,180].

Lipid profiling has been applied to plasma collected from patients with IPF. The analysis of the lipid species found in plasma from IPF patients compared with control patients identified a six-lipid signature, including triglycerides and PCs (Figure 2), that could be used to differentiate samples from the two groups [181]. An in-depth lipidomic analysis showed significantly increased triglycerides and PCs in patients with stable vs. rapidly progressing IPF [182]. In the progressor group, the enriched lipidomic networks were the metabolism of linoleic acid, arachidonic acid, and glycerophospholipids.

Although these data from clinical trials are appealing, they need to be challenged in larger and multi-centric cohorts before being potentially used in clinical practice. The heterogeneity of patients diagnosed with IPF will probably represent a major bottleneck in this quest to use a lipid signature to evaluate prognosis or disease progression. Indeed, IPF is often diagnosed in an aged population that likely brings other conditions (and accompanying medication) on top of the disease itself. In particular, the challenge here would come from patients with lipid-lowering drugs (statins, fibrates). Moreover, additional research is needed to rule out the possibility of increased arachidonic acid metabolism due to inflammation, which could be observed during episodes of acute IPF exacerbation. 

## 6. Lipid-Focused Therapies

Lipids are essential components of cellular metabolism within all cells. Consequently, several approaches focused on controlling cell metabolism, including lipid regulation, have been investigated for their anti-fibrotic properties. Metformin, which is used clinically in diabetes, can reverse pulmonary fibrosis after bleomycin administration [183]. In IPF fibroblasts, metformin promotes lipid accumulation and metabolism with enhanced PPAR-γ [11]. In parallel, reduced TGF-β1-mediated activation and collagen production are observed in these cells after exposure to metformin, consistent with a myofibroblast differentiation towards a lipofibroblast phenotype [11]. In addition to cell metabolism, senescence is a major hallmark of IPF, and senolytic drugs are actively being studied as a therapy for fibrotic disorders [5]. The senolytic drug quercetin has a beneficial effect on bleomycin-induced pulmonary fibrosis in mice [184]. Interestingly, quercetin administration has been associated with a decreased S1P signaling in fibroblasts [184]. Another active area of investigation is S1P signaling. Chemical antagonists of S1P receptor 2 have been tested in mice exposed to bleomycin [185]. In this model, when compared to controls, the S1P receptor 2 inhibition seemed to diminish fibrosis as effectively as the anti-fibrotic drug pirfenidone [186].

As described above, increased levels of HDL are associated with less severe disease in patients with IPF [177], suggesting that cholesterol accumulation may be detrimental. Over the last decades, a growing number of cholesterol-lowering drugs have been developed, statins being one of them. The administration of statins seems to have a beneficial effect on bleomycin-induced lung fibrosis in rats [185]. This observation concurs with retrospective studies assessing the effect of statins in patients with IPF [187,188].

Disturbed lipid and fatty acid levels appear to be a key component of fibrosis. Many studies have therefore tried to correct the levels of lipids that decrease during fibrosis. Supplementation with anti-oxidative lipids such as α-lipoic acid decreases fibrosis induced by bleomycin [189]. The effect of the omega-3 fatty acid DHA has also been tested in pre-clinical models. Compared with controls, mice subjected to bleomycin and receiving the protectin DX (a DHA derivative) display reduced fibrosis with enhanced lung function and a longer life span [190]. Protectin DX’s mechanistic ability to reverse fibrosis has been observed, explaining the anti-fibrotic properties of the compound. In vitro, fibroblasts cultured in the presence of the DHA derivative maresin 1 exhibit fewer activation markers upon TGF-β exposure, in addition to decreased α-SMA expression, Smad signaling, and ERK signaling [191]. In mice exposed to bleomycin, the local administration of DHA during the early inflammation phase inhibits collagen accumulation [192]. DHA is associated with decreased levels of eicosanoid production in this model. 

Eicosanoid imbalance and further leukotriene production over prostaglandin is believed to be a dysregulated mechanism that has an important role in fibrosis progression. Leukotriene inhibitors have already been assessed in pre-clinical models of pulmonary fibrosis and were found to decrease fibrosis in mice when administered starting on D7 post bleomycin exposure [193]. Similarly, agonists of the anti-inflammatory lipoxin receptors inhibit collagen accumulation following bleomycin exposition [194].

LPA is another extracellular bioactive lipid with pro-fibrotic activity. Pharmacological inhibition of LPA or autotaxin, which produces LPA, is an active area of research. In the bleomycin model, inhibitors of autotaxin or LAP receptors protect mice from fibrosis [195]. Thanks to encouraging data from pre-clinical models, autotaxin inhibitors are being tested in patients with IPF [196]. 

Lipids can also serve as extracellular mediators that activate cellular signaling by binding to surface receptors such as fatty acid receptor CD36. Recently, this receptor and its inhibition have gained significant attention in the field of cancer as a potential strategy to limit metastasis [197]. CD36 expression correlates with high EMT and poor prognosis in patients with cancer [198]. Its function in lipid-mediated cell reprogramming (EMT) and immune cell activation has led to the testing of CD36 inhibitors in clinical trials in patients with cancer [199]. In silica-induced lung fibrosis in rats, lentivirus-based CD36 silencing diminished hydroxyproline content and other fibrosis-related markers compared to controls [200]. The potential of CD36 inhibitors, while developed in cancer, remains to be investigated in fibrosis

As discussed above, cellular communication is of major importance in IPF, and part of this communication is mediated through EVs [201,202]. There is growing interest regarding the use of EVs isolated from anti-inflammatory and anti-fibrotic mesenchymal stem cells as a therapy for fibrotic disorders [203,204]. Such strategies have been employed in preclinical models and seem to be efficient in reducing experimental lung fibrosis [203,205,206]. These data exist, but more research is required to fully characterize these vesicles and develop production methods for translational studies.

Nevertheless, numerous lipid-targeting strategies have been tested in patients with IPF in line with these preclinical data. The autotoxin inhibitor GLPG190 was tested in a phase 3 study in IPF but was stopped because the benefit-risk profile no longer supported the continuation of the study (NCT03733444). Moreover, high-affinity LPA1 antagonists were assessed vs placebo in a phase 2 study in patients with IPF and found to significantly reduce FVC decline [207]. They are currently being tested in patients with IPF or progressive fibrotic interstitial lung disease (ClinicalTrials.gov Identifier: NCT04308681). Other lipid-targeting drugs are currently being tested in IPF, such as the PBI-4050 compound, which completed an open-label phase 2 clinical trial in IPF [208,209]. PBI-4050 is known to modulate the activity of G-protein coupled lipid receptors GPR40 and GPR84. This molecule was tested alone or in combination with either pirfenidone or nintedanib. The published results from this trial show no significant change in lung function (%FVC) from baseline to three months in patients with PBI-4050 alone or PBI-4050 + nintedanib [208]. 

## Figures and Tables

**Table 1:** Lipid metabolism dysregulation during pulmonary fibrosis.

Proteins Associated with Lipid Metabolism	Regulation in Lung Fibrosis	Evidence	Disease/Model	Cell Type(s)	Reference
ATP Binding Cassette Subfamily A Member 3	down	scRNAseq	patient, bleomycin model	ATII	[99]
Sterol Regulatory Element Binding Transcription Factor 2	up	scRNAseq	bleomycin model	ATII, lipofibroblasts	[102]
Peroxisome Proliferator Activated Receptor Gamma	down	qPCR	IPF	lung tissue	[103]
Elongation of Long Chain Fatty Acids 6	down	qPCR, IHC	patient, bleomycin model	ATII	[104]
Autotaxin	up	IHC, qPCR, ELISA	patient, bleomycin model	hyperplastic bronchiolar and alveolar epithelium, fibroblasts, macrophages	[105]
Arachidonate 5-Lipoxygenase	up	qPCR	bleomycin model	senescent cells	[106]
Leukotriene C4 Synthase	up	qPCR	bleomycin model	senescent cells	[106]
Prostaglandin D2 Synthase	up	qPCR	bleomycin model	senescent cells	[106]
Prostaglandin-Endoperoxide Synthase 2	up	qPCR	bleomycin model	senescent cells	[106]
Prostaglandin E Synthase	up	qPCR	bleomycin model	senescent cells	[106]
Prostaglandin E Receptor 2	down	western blot	IPF	fibroblasts	[107]
Prostaglandin E Synthase	down	IHC	IPF	epithelial cells, fibroblasts	[108]
Sphingosine-1-Phosphate Lyase 1	up	IHC, western blot, qPCR	patient, bleomycin model	fibrotic tissue, fibroblasts, PBMCs	[109]

## References

[1] Lederer D.J., Martinez F.J. (2018). Idiopathic pulmonary fibrosis. N. Engl. J. Med..

[2] Richeldi L., Cottin V., du Bois R.M., Selman M., Kimura T., Bailes Z., Schlenker-Herceg R., Stowasser S., Brown K.K. (2016). Nintedanib in patients with idiopathic pulmonary fibrosis: Combined evidence from the TOMORROW and INPULSIS(®) trials. Respir. Med..

[3] King T.E., Bradford W.Z., Castro-Bernardini S., Fagan E.A., Glaspole I., Glassberg M.K., Gorina E., Hopkins P.M., Kardatzke D., Lancaster L. (2014). A phase 3 trial of pirfenidone in patients with idiopathic pulmonary fibrosis. N. Engl. J. Med..

[4] Fernandez I.E., Eickelberg O. (2012). New cellular and molecular mechanisms of lung injury and fibrosis in idiopathic pulmonary fibrosis. Lancet.

[5] Burgy O., Bellaye P.-S., Beltramo G., Goirand F., Bonniaud P. (2020). Pathogenesis of fibrosis in interstitial lung disease. Curr. Opin. Pulm. Med..

[6] Phan T.H.G., Paliogiannis P., Nasrallah G.K., Giordo R., Eid A.H., Fois A.G., Zinellu A., Mangoni A.A., Pintus G. (2021). Emerging cellular and molecular determinants of idiopathic pulmonary fibrosis. Cell. Mol. Life Sci..

[7] Strunz M., Simon L.M., Ansari M., Kathiriya J.J., Angelidis I., Mayr C.H., Tsidiridis G., Lange M., Mattner L.F., Yee M. (2020). Alveolar regeneration through a Krt8+ transitional stem cell state that persists in human lung fibrosis. Nat. Commun..

[8] Jiang P., Gil de Rubio R., Hrycaj S.M., Gurczynski S.J., Riemondy K.A., Moore B.B., Omary M.B., Ridge K.M., Zemans R.L. (2020). Ineffectual type 2-to-type 1 alveolar epithelial cell differentiation in idiopathic pulmonary fibrosis: Persistence of the KRT8hi transitional state. Am. J. Respir. Crit. Care Med..

[9] Kathiriya J.J., Wang C., Zhou M., Brumwell A., Cassandras M., Le Saux C.J., Cohen M., Alysandratos K.-D., Wang B., Wolters P. (2022). Human alveolar type 2 epithelium transdifferentiates into metaplastic KRT5+ basal cells. Nat. Cell. Biol..

[10] Tsukui T., Sun K.-H., Wetter J.B., Wilson-Kanamori J.R., Hazelwood L.A., Henderson N.C., Adams T.S., Schupp J.C., Poli S.D., Rosas I.O. (2020). Collagen-producing lung cell atlas identifies multiple subsets with distinct localization and relevance to fibrosis. Nat. Commun..

[11] Kheirollahi V., Wasnick R.M., Biasin V., Vazquez-Armendariz A.I., Chu X., Moiseenko A., Weiss A., Wilhelm J., Zhang J.-S., Kwapiszewska G. (2019). Metformin induces lipogenic differentiation in myofibroblasts to reverse lung fibrosis. Nat. Commun..

[12] Xie T., Wang Y., Deng N., Huang G., Taghavifar F., Geng Y., Liu N., Kulur V., Yao C., Chen P. (2018). Single-cell deconvolution of fibroblast heterogeneity in mouse pulmonary fibrosis. Cell Rep..

[13] Kim K.K., Kugler M.C., Wolters P.J., Robillard L., Galvez M.G., Brumwell A.N., Sheppard D., Chapman H.A. (2006). Alveolar epithelial cell mesenchymal transition develops in vivo during pulmonary fibrosis and is regulated by the extracellular matrix—PubMed. Proc. Natl. Acad. Sci. USA.

[14] Rock J.R., Barkauskas C.E., Cronce M.J., Xue Y., Harris J.R., Liang J., Noble P.W., Hogan B.L. (2011). Multiple stromal populations contribute to pulmonary fibrosis without evidence for epithelial to mesenchymal transition—PubMed. Proc. Natl. Acad. Sci. USA.

[15] Decologne N., Kolb M., Margetts P.J., Menetrier F., Artur Y., Garrido C., Gauldie J., Camus P., Bonniaud P. (2007). TGF-Beta1 induces progressive pleural scarring and subpleural Fibrosis—PubMed. J. Immunol..

[16] Hashimoto N., Phan S.H., Imaizumi K., Matsuo M., Nakashima H., Kawabe T., Shimokata K., Hasegawa Y. (2010). Endothelial-mesenchymal transition in bleomycin-induced pulmonary fibrosis—PubMed. Am. J. Respir. Cell Mol. Biol..

[17] Jia W., Wang Z., Gao C., Wu J., Wu Q. (2021). Trajectory modeling of endothelial-to-mesenchymal transition reveals galectin-3 as a mediator in pulmonary fibrosis. Cell Death Dis..

[18] Lim W.W., Corden B., Ng B., Vanezis K., D’Agostino G., Widjaja A.A., Song W.H., Xie C., Su L., Kwek X.Y. (2020). Interleukin-11 is important for vascular smooth muscle phenotypic switching and aortic inflammation, fibrosis and remodeling in mouse models—PubMed. Sci. Rep..

[19] Fois A.G., Posadino A.M., Giordo R., Cossu A., Agouni A., Rizk N.M., Pirina P., Carru C., Zinellu A., Pintus G. (2018). Antioxidant Activity Mediates Pirfenidone Antifibrotic Effects in Human Pulmonary Vascular Smooth Muscle Cells Exposed to Sera of Idiopathic Pulmonary Fibrosis Patients. Oxidative Med. Cell. Longev..

[20] Reyfman P.A., Walter J.M., Joshi N., Anekalla K.R., McQuattie-Pimentel A.C., Chiu S., Fernandez R., Akbarpour M., Chen C.-I., Ren Z. (2019). Single-cell transcriptomic analysis of human lung provides insights into the pathobiology of pulmonary fibrosis. Am. J. Respir. Crit. Care Med..

[21] Morse C., Tabib T., Sembrat J., Buschur K.L., Bittar H.T., Valenzi E., Jiang Y., Kass D.J., Gibson K., Chen W. (2019). Proliferating SPP1/MERTK-expressing macrophages in idiopathic pulmonary fibrosis. Eur. Respir. J..

[22] Adams T.S., Schupp J.C., Poli S., Ayaub E.A., Neumark N., Ahangari F., Chu S.G., Raby B.A., DeIuliis G., Januszyk M. (2020). Single-Cell RNA-seq reveals ectopic and aberrant lung-resident cell populations in idiopathic pulmonary fibrosis. Sci. Adv..

[23] Misharin A.V., Morales-Nebreda L., Reyfman P.A., Cuda C.M., Walter J.M., McQuattie-Pimentel A.C., Chen C.-I., Anekalla K.R., Joshi N., Williams K.J.N. (2017). Monocyte-derived alveolar macrophages drive lung fibrosis and persist in the lung over the life span. J. Exp. Med..

[24] Mould K.J., Moore C.M., McManus S.A., McCubbrey A.L., McClendon J.D., Griesmer C.L., Henson P.M., Janssen W.J. (2021). Airspace macrophages and monocytes exist in transcriptionally distinct subsets in healthy adults. Am. J. Respir. Crit. Care Med..

[25] Schiller H.B., Mayr C.H., Leuschner G., Strunz M., Staab-Weijnitz C., Preisendörfer S., Eckes B., Moinzadeh P., Krieg T., Schwartz D.A. (2017). Deep proteome profiling reveals common prevalence of MZB1-positive plasma B cells in human lung and skin fibrosis. Am. J. Respir. Crit. Care Med..

[26] Mould K.J., Jackson N.D., Henson P.M., Seibold M., Janssen W.J. (2019). Single cell RNA sequencing identifies unique inflammatory airspace macrophage subsets. JCI Insight.

[27] McQuattie-Pimentel A.C., Ren Z., Joshi N., Watanabe S., Stoeger T., Chi M., Lu Z., Sichizya L., Aillon R.P., Chen C.-I. (2021). The lung microenvironment shapes a dysfunctional response of alveolar macrophages in aging. J. Clin. Investig..

[28] Hackett T.L., Osei E.T. (2021). Modeling extracellular matrix-cell interactions in lung repair and chronic disease. Cells.

[29] Burgy O., Königshoff M. (2018). The WNT signaling pathways in wound healing and fibrosis. Matrix Biol..

[30] Sime P.J., Xing Z., Graham F.L., Csaky K.G., Gauldie J. (1997). Adenovector-mediated gene transfer of active transforming growth factor-beta1 induces prolonged severe fibrosis in rat lung—PubMed. J. Clin. Investig..

[31] Bonniaud P., Margetts P.J., Kolb M., Schroeder J.A., Kapoun A.M., Damm D., Murphy A., Chakravarty S., Dugar S., Higgins L. (2005). Progressive transforming growth factor beta1-induced lung fibrosis is blocked by an orally active ALK5 kinase inhibitor. Am. J. Respir. Crit. Care Med..

[32] Nabhan A.N., Brownfield D.G., Harbury P.B., Krasnow M.A., Desai T.J. (2018). Single-cell Wnt signaling niches maintain stemness of alveolar type 2 cells. Science.

[33] Liu F., Lagares D., Choi K.M., Stopfer L., Marinković A., Vrbanac V., Probst C.K., Hiemer S.E., Sisson T.H., Horowitz J.C. (2015). Mechanosignaling through YAP and TAZ drives fibroblast activation and fibrosis. Am. J. Physiol. Lung Cell. Mol. Physiol..

[34] Sun T., Huang Z., Zhang H., Posner C., Jia G., Ramalingam T.R., Xu M., Brightbill H., Egen J.G., Dey A. (2019). TAZ is required for lung alveolar epithelial cell differentiation after injury. JCI Insight.

[35] Meiners S., Eickelberg O., Königshoff M. (2015). Hallmarks of the ageing lung. Eur. Respir. J..

[36] Schafer M.J., White T.A., Iijima K., Haak A.J., Ligresti G., Atkinson E.J., Oberg A.L., Birch J., Salmonowicz H., Zhu Y. (2017). Cellular senescence mediates fibrotic pulmonary disease. Nat. Commun..

[37] Lehmann M., Korfei M., Mutze K., Klee S., Skronska-Wasek W., Alsafadi H.N., Ota C., Costa R., Schiller H.B., Lindner M. (2017). Senolytic drugs target alveolar epithelial cell function and attenuate experimental lung fibrosis ex vivo. Eur. Respir. J..

[38] Kellogg D.L., Musi N., Nambiar A.M. (2021). Cellular Senescence in Idiopathic Pulmonary Fibrosis. Curr. Mol. Biol. Rep..

[39] Justice J.N., Nambiar A.M., Tchkonia T., LeBrasseur N.K., Pascual R., Hashmi S.K., Prata L., Masternak M.M., Kritchevsky S.B., Musi N. (2019). Senolytics in idiopathic pulmonary fibrosis: Results from a first-in-human, open-label, pilot study. EBioMedicine.

[40] Bellaye P.-S., Beltramo G., Burgy O., Collin B., Cochet A., Bonniaud P. (2022). Measurement of hypoxia in the lung in idiopathic pulmonary fibrosis: A matter of control. Eur. Respir. J..

[41] Porter J.C., Win T., Erlandsson K., Fraioli F., Rashidnasab A., Holman B., Ganeshan B., Screaton N.J., Maher T.M., Endozo R. (2021). Measurement of hypoxia in the lung in idiopathic pulmonary fibrosis: An F-MISO PET/CT study. Eur. Respir. J..

[42] Tanguy J., Goirand F., Bouchard A., Frenay J., Moreau M., Mothes C., Oudot A., Helbling A., Guillemin M., Bonniaud P. (2021). [18F]FMISO PET/CT imaging of hypoxia as a non-invasive biomarker of disease progression and therapy efficacy in a preclinical model of pulmonary fibrosis: Comparison with the [18F]FDG PET/CT approach. Eur. J. Pediatr..

[43] Gille T., Didier M., Boubaya M., Moya L., Sutton A., Carton Z., Baran-Marszak F., Sadoun-Danino D., Israël-Biet D., Cottin V. (2017). Obstructive sleep apnoea and related comorbidities in incident idiopathic pulmonary fibrosis. Eur. Respir. J..

[44] Gille T., Didier M., Rotenberg C., Delbrel E., Marchant D., Sutton A., Dard N., Haine L., Voituron N., Bernaudin J.-F. (2018). Intermittent Hypoxia Increases the Severity of Bleomycin-Induced Lung Injury in Mice. Oxidative Med. Cell. Longev..

[45] Agudelo C.W., Samaha G., Garcia-Arcos I. (2020). Alveolar Lipids in Pulmonary Disease. A Review. Lipids Health Dis..

[46] Castillo-Sánchez J.C., Cruz A., Pérez-Gil J. (2021). Structural hallmarks of lung surfactant: Lipid-protein interactions, membrane structure and future challenges. Arch. Biochem. Biophys..

[47] Nuñez-Garcia M., Gomez-Santos B., Buqué X., García-Rodriguez J.L., Romero M.R., Marin J.J.G., Arteta B., García-Monzón C., Castaño L., Syn W.-K. (2017). Osteopontin regulates the cross-talk between phosphatidylcholine and cholesterol metabolism in mouse liver. J. Lipid Res..

[48] Yan X., Wang J., Zhu Y., Feng W., Zhai C., Liu L., Shi W., Wang Q., Zhang Q., Chai L. (2019). S1P induces pulmonary artery smooth muscle cell proliferation by activating calcineurin/NFAT/OPN signaling pathway. Biochem. Biophys. Res. Commun..

[49] Romani P., Brian I., Santinon G., Pocaterra A., Audano M., Pedretti S., Mathieu S., Forcato M., Bicciato S., Manneville J.-B. (2019). Extracellular matrix mechanical cues regulate lipid metabolism through Lipin-1 and SREBP. Nat. Cell Biol..

[50] Ho L.T.Y., Skiba N., Ullmer C., Rao P.V. (2018). Lysophosphatidic Acid Induces ECM Production via Activation of the Mechanosensitive YAP/TAZ Transcriptional Pathway in Trabecular Meshwork Cells. Investig. Opthalmol. Vis. Sci..

[51] Mamazhakypov A., Schermuly R.T., Schaefer L., Wygrecka M. (2019). Lipids—Two sides of the same coin in lung fibrosis. Cell Signal..

[52] Suryadevara V., Ramchandran R., Kamp D.W., Natarajan V. (2020). Lipid mediators regulate pulmonary fibrosis: Potential mechanisms and signaling pathways. Int. J. Mol. Sci..

[53] Hollenbeck C.B. (2012). An Introduction to the Nutrition and Metabolism of Choline. Cent. Nerv. Syst. Agents Med. Chem..

[54] Ridgway N.D. (2013). The role of phosphatidylcholine and choline metabolites to cell proliferation and survival. Crit. Rev. Biochem. Mol. Biol..

[55] Zeisel S.H. (1996). Choline. A nutrient that is involved in the regulation of cell proliferation, cell death, and cell transformation. Adv. Exp. Med. Biol..

[56] Hirai K., Watanabe S., Nishijima N., Shibata K., Hase A., Yamanaka T., Inazu M. (2020). Molecular and functional analysis of choline transporters and antitumor effects of choline transporter-like protein 1 inhibitors in human pancreatic cancer cells. Int. J. Mol. Sci..

[57] Inazu M., Yamada T., Kubota N., Yamanaka T. (2013). Functional expression of choline transporter-like protein 1 (CTL1) in small cell lung carcinoma cells: A target molecule for lung cancer therapy. Pharmacol. Res..

[58] Wang B., Tontonoz P. (2019). Phospholipid remodeling in physiology and disease. Annu. Rev. Physiol..

[59] Gibellini F., Smith T.K. (2010). The Kennedy pathway - De novo synthesis of phosphatidylethanolamine and phosphatidylcholine. IUBMB Life.

[60] Ishiguro N., Oyabu M., Sato T., Maeda T., Minami H., Tamai I. (2008). Decreased biosynthesis of lung surfactant constituent phosphatidylcholine due to inhibition of choline transporter by gefitinib in lung alveolar cells. Pharm. Res..

[61] Dodia C., Fisher A.B., Chander A., Kleinzeller A. (1992). Inhibitors of choline transport in alveolar type ii epithelial cells. Am. J. Respir. Cell Mol. Biol..

[62] Lockman P.R., Allen D.D. (2002). The transport of choline. Drug Dev. Ind. Pharm..

[63] Oelberg D.G., Xu F. (1998). Conductive choline transport by alveolar epithelial plasma membrane vesicles. Mol. Genet. Metab..

[64] Agostoni C., Bruzzese M.G. (1992). Fatty acids: Their biochemical and functional classification. Pediatr. Med. Chir..

[65] Tvrzicka E., Kremmyda L.-S., Stankova B., Zak A. (2011). Fatty acids as biocompounds: Their role in human metabolism, health and disease—A review. Part 1: Classification, dietary sources and biological functions. Biomed. Pap. Med. Fac. Univ. Palacky Olomouc Czech Repub..

[66] Beld J., Lee D.J., Burkart M.D. (2015). Fatty acid biosynthesis revisited: Structure elucidation and metabolic engineering. Mol. Biosyst..

[67] Ohno Y., Suto S., Yamanaka M., Mizutani Y., Mitsutake S., Igarashi Y., Sassa T., Kihara A. (2010). ELOVL1 production of C24 Acyl-CoAs is linked to C24 sphingolipid synthesis. Proc. Natl. Acad. Sci. USA.

[68] Guillou H., Zadravec D., Martin P.G.P., Jacobsson A. (2010). The key roles of elongases and desaturases in mammalian fatty acid metabolism: Insights from transgenic mice. Prog. Lipid Res..

[69] Jakobsson A., Westerberg R., Jacobsson A. (2006). Fatty acid elongases in mammals: Their regulation and roles in metabolism. Prog. Lipid Res..

[70] Bach L., Faure J.-D. (2010). Role of very-long-chain fatty acids in plant development, when chain length does matter. Comptes Rendus. Biol..

[71] McMaster C.R. (2018). From yeast to humans—Roles of the Kennedy pathway for phosphatidylcholine synthesis. FEBS Lett..

[72] Perrakis A., Moolenaar W.H. (2014). Autotaxin: Structure-function and signaling. J. Lipid Res..

[73] Song J., Clair T., Noh J.H., Eun J.W., Ryu S.Y., Lee S.N., Ahn Y.M., Kim S.Y., Lee S.H., Park W.S. (2005). Autotaxin (LysoPLD/NPP2) protects fibroblasts from apoptosis through its enzymatic product, lysophosphatidic acid, utilizing albumin-bound substrate. Biochem. Biophys. Res. Commun..

[74] Onono F.O., Morris A.J. (2020). Phospholipase D and choline metabolism. Handb. Exp. Pharmacol..

[75] Wang B., Wu L., Chen J., Dong L., Chen C., Wen Z., Hu J., Fleming I., Wang D.W. (2021). Metabolism pathways of arachidonic acids: Mechanisms and potential therapeutic targets. Signal. Transduct. Target. Ther..

[76] Peters-Golden M., Henderson W.R. (2007). Leukotrienes. N. Engl. J. Med..

[77] Schildknecht S., Daiber A., Ghisla S., Cohen R.A., Bachschmid M.M. (2007). Acetaminophen inhibits prostanoid synthesis by scavenging the PGHS-activator peroxynitrite. FASEB J..

[78] Moore B.B., Peters-Golden M. (2006). Opposing roles of leukotrienes and prostaglandins in fibrotic lung disease. Expert Rev. Clin. Immunol..

[79] Chandrasekharan J.A., Sharma-Walia N. (2015). Lipoxins: Nature’s way to resolve inflammation. J. Inflamm. Res..

[80] Beers M.F., Mulugeta S. (2017). The biology of the ABCA3 lipid transporter in lung health and disease. Cell Tissue Res..

[81] Zhao L., Li Y., Ding Q., Li Y., Chen Y., Ruan X.Z. (2021). CD36 senses dietary lipids and regulates lipids homeostasis in the intestine. Front. Physiol..

[82] Silverstein R.L., Febbraio M. (2009). CD36, a scavenger receptor involved in immunity, metabolism, angiogenesis, and behavior. Sci. Signal..

[83] Xu S., Chaudhary O., Rodríguez-Morales P., Sun X., Chen D., Zappasodi R., Xu Z., Pinto A.F., Williams A., Schulze I. (2021). Uptake of oxidized lipids by the scavenger receptor CD36 promotes lipid peroxidation and dysfunction in CD8+ T cells in tumors. Immunity.

[84] Banesh S., Trivedi V. (2020). Therapeutic potentials of scavenger receptor CD36 Mediated innate immune responses against infectious and non-infectious diseases. Curr. Drug. Discov. Technol..

[85] Dobri A.-M., Dudău M., Enciu A.-M., Hinescu M.E. (2021). CD36 in Alzheimer’s disease: An overview of molecular mechanisms and therapeutic targeting. Neuroscience.

[86] Ogretmen B. (2018). Sphingolipid metabolism in cancer signalling and therapy. Nat. Rev. Cancer.

[87] Gomez-Larrauri A., Presa N., Dominguez-Herrera A., Ouro A., Trueba M., Gomez-Muñoz A. (2020). Role of bioactive sphingolipids in physiology and pathology. Essays Biochem..

[88] Levy M., Futerman A.H. (2010). Mammalian ceramide synthases. IUBMB Life.

[89] Mullen T.D., Hannun Y.A., Obeid L.M. (2012). Ceramide synthases at the centre of sphingolipid metabolism and biology. Biochem. J..

[90] Wang E., He X., Zeng M. (2018). The Role of S1P and the related signaling pathway in the development of tissue fibrosis. Front. Pharmacol..

[91] Cuvillier O. (2012). Sphingosine 1-phosphate receptors: From biology to physiopathology. Med. Sci..

[92] Cuvillier O. (2002). Sphingosine in apoptosis signaling. Biochim. Biophys. Acta.

[93] Brizuela L., Ader I., Mazerolles C., Bocquet M., Malavaud B., Cuvillier O. (2012). First evidence of sphingosine 1-phosphate lyase protein expression and activity downregulation in human neoplasm: Implication for resistance to therapeutics in prostate cancer. Mol. Cancer Ther..

[94] Ceccom J., Loukh N., Lauwers-Cances V., Touriol C., Nicaise Y., Gentil C., Uro-Coste E., Pitson S., Maurage C.A., Duyckaerts C. (2014). Reduced sphingosine kinase-1 and enhanced sphingosine 1-phosphate lyase expression demonstrate deregulated sphingosine 1-phosphate signaling in Alzheimer’s disease. Acta Neuropathol. Commun..

[95] Holthuis J., Menon A. (2014). Lipid landscapes and pipelines in membrane homeostasis. Nature.

[96] Endo A. (2010). A historical perspective on the discovery of statins. Proc. Jpn. Acad. Ser. B Phys. Biol. Sci..

[97] Elshourbagy N.A., Meyers H.V., Abdel-Meguid S.S. (2014). Cholesterol: The good, the bad, and the ugly—Therapeutic targets for the treatment of dyslipidemia. Med. Princ. Pract..

[98] Summer R., Mora A.L. (2019). Lipid metabolism: A new player in the conundrum of lung fibrosis. Am. J. Respir. Cell Mol. Biol..

[99] Xu Y., Mizuno T., Sridharan A., Du Y., Guo M., Tang J., Wikenheiser-Brokamp K.A., Perl A.-K.T., Funari V.A., Gokey J.J. (2016). Single-Cell RNA sequencing identifies diverse roles of epithelial cells in idiopathic pulmonary fibrosis. JCI Insight.

[100] Manali E.D., Legendre M., Nathan N., Kannengiesser C., Coulomb-L’Hermine A., Tsiligiannis T., Tomos P., Griese M., Borie R., Clement A. (2019). Bi-allelic missense ABCA3 mutations in a patient with childhood ILD who reached adulthood. ERJ Open Res..

[101] Romero F., Shah D., Duong M., Penn R.B., Fessler M.B., Madenspacher J., Stafstrom W., Kavuru M., Lu B., Kallen C.B. (2015). A pneumocyte-macrophage paracrine lipid axis drives the lung toward fibrosis. Am. J. Respir. Cell Mol. Biol..

[102] Angelidis I., Simon L.M., Fernandez I.E., Strunz M., Mayr C.H., Greiffo F.R., Tsitsiridis G., Ansari M., Graf E., Strom T.-M. (2019). An atlas of the aging lung mapped by single cell transcriptomics and deep tissue proteomics. Nat. Commun..

[103] El Agha E., Moiseenko A., Kheirollahi V., De Langhe S., Crnkovic S., Kwapiszewska G., Szibor M., Kosanovic D., Schwind F., Schermuly R.T. (2017). Two-way conversion between lipogenic and myogenic fibroblastic phenotypes marks the progression and resolution of lung fibrosis. Cell Stem Cell.

[104] Sunaga H., Matsui H., Ueno M., Maeno T., Iso T., Syamsunarno M.R.A.A., Anjo S., Matsuzaka T., Shimano H., Yokoyama T. (2013). Deranged fatty acid composition causes pulmonary fibrosis in Elovl6-deficient mice. Nat. Commun..

[105] Oikonomou N., Mouratis M.-A., Tzouvelekis A., Kaffe E., Valavanis C., Vilaras G., Karameris A., Prestwich G.D., Bouros D., Aidinis V. (2012). Pulmonary autotaxin expression contributes to the pathogenesis of pulmonary fibrosis. Am. J. Respir. Cell Mol. Biol..

[106] Wiley C.D., Brumwell A.N., Davis S.S., Jackson J.R., Valdovinos A., Calhoun C., Alimirah F., Castellanos C.A., Ruan R., Wei Y. (2019). Secretion of leukotrienes by senescent lung fibroblasts promotes pulmonary fibrosis. JCI Insight.

[107] Huang S.K., Wettlaufer S.H., Hogaboam C.M., Flaherty K.R., Martinez F.J., Myers J.L., Colby T.V., Travis W.D., Toews G.B., Peters-Golden M. (2008). Variable prostaglandin E2 resistance in fibroblasts from patients with usual interstitial pneumonia. Am. J. Respir. Crit. Care Med..

[108] Berhan A., Harris T., Jaffar J., Jativa F., Langenbach S., Lönnstedt I., Alhamdoosh M., Ng M., Lee P., Westall G. (2020). Cellular microenvironment stiffness regulates eicosanoid production and signaling pathways. Am. J. Respir. Cell Mol. Biol..

[109] Huang L.S., Berdyshev E.V., Tran J.T., Xie L., Chen J., Ebenezer D.L., Mathew B., Gorshkova I., Zhang W., Reddy S.P. (2015). Sphingosine-1-phosphate lyase is an endogenous suppressor of pulmonary fibrosis: Role of S1P signalling and autophagy. Thorax.

[110] Zemski Berry K.A., Murphy R.C., Kosmider B., Mason R.J. (2017). Lipidomic characterization and localization of phospholipids in the human lung. J. Lipid Res..

[111] Saito K., Tanaka N., Ikari J., Suzuki M., Anazawa R., Abe M., Saito Y., Tatsumi K. (2019). Comprehensive lipid profiling of bleomycin-induced lung injury. J. Appl. Toxicol..

[112] Kulkarni Y.M., Dutta S., Iyer A.K.V., Wright C.A., Ramesh V., Kaushik V., Semmes O.J., Azad N. (2018). A lipidomics approach to identifying key lipid species involved in VEGF-inhibitor mediated attenuation of bleomycin-induced pulmonary fibrosis. Proteom. Clin. Appl..

[113] Chu S.G., Villalba J.A., Liang X., Xiong K., Tsoyi K., Ith B., Ayaub E.A., Tatituri R.V., Byers D.E., Hsu F.-F. (2019). Palmitic acid-rich high-fat diet exacerbates experimental pulmonary fibrosis by modulating endoplasmic reticulum stress. Am. J. Respir. Cell Mol. Biol..

[114] Kim H.-S., Yoo H.J., Lee K.M., Song H.E., Kim S.J., Lee J.O., Hwang J.J., Song J.W. (2021). Stearic acid attenuates profibrotic signalling in idiopathic pulmonary fibrosis. Respirology.

[115] Chung K.-P., Hsu C.-L., Fan L.-C., Huang Z., Bhatia D., Chen Y.-J., Hisata S., Cho S.J., Nakahira K., Imamura M. (2019). Mitofusins regulate lipid metabolism to mediate the development of lung fibrosis. Nat. Commun..

[116] Romero F., Hong X., Shah D., Kallen C.B., Rosas I., Guo Z., Schriner D., Barta J., Shaghaghi H., Hoek J.B. (2018). Lipid synthesis is required to resolve endoplasmic reticulum stress and limit fibrotic responses in the lung. Am. J. Respir. Cell Mol. Biol..

[117] Kikuchi T., Sugiura H., Koarai A., Ichikawa T., Minakata Y., Matsunaga K., Nakanishi M., Hirano T., Akamatsu K., Yanagisawa S. (2012). Increase of 27-hydroxycholesterol in the airways of patients with COPD: Possible role of 27-hydroxycholesterol in tissue fibrosis. Chest.

[118] Ichikawa T., Sugiura H., Koarai A., Kikuchi T., Hiramatsu M., Kawabata H., Akamatsu K., Hirano T., Nakanishi M., Matsunaga K. (2013). 25-hydroxycholesterol promotes fibroblast-mediated tissue remodeling through NF-ΚB dependent pathway. Exp. Cell Res..

[119] Yao X., Gordon E.M., Figueroa D.M., Barochia A.V., Levine S.J. (2016). Emerging roles of apolipoprotein E and apolipoprotein A-I in the pathogenesis and treatment of lung disease. Am. J. Respir. Cell Mol. Biol..

[120] Yu Z., Jin J., Wang Y., Sun J. (2017). High density lipoprotein promoting proliferation and migration of type II alveolar epithelial cells during inflammation state. Lipids Health Dis..

[121] Pison U., Max M., Neuendank A., Weissbach S., Pietschmann S. (1994). Host defence capacities of pulmonary surfactant: Evidence for “non-Surfactant” functions of the surfactant system. Eur. J. Clin. Investig..

[122] Gille C., Spring B., Bernhard W., Gebhard C., Basile D., Lauber K., Poets C.F., Orlikowsky T.W. (2007). Differential effect of surfactant and its saturated phosphatidylcholines on human blood macrophages. J. Lipid Res..

[123] Griese M., Bonella F., Costabel U., de Blic J., Tran N.-B., Liebisch G. (2019). Quantitative lipidomics in pulmonary alveolar proteinosis. Am. J. Respir. Crit. Care Med..

[124] Esteves P., Blanc L., Celle A., Dupin I., Maurat E., Amoedo N., Cardouat G., Ousova O., Gales L., Bellvert F. (2021). Crucial role of fatty acid oxidation in asthmatic bronchial smooth muscle remodelling. Eur. Respir. J..

[125] Balijepalli P., Sitton C.C., Meier K.E. (2021). Lysophosphatidic acid signaling in cancer cells: What makes LPA so special?. Cells.

[126] Tager A.M., LaCamera P., Shea B.S., Campanella G.S., Selman M., Zhao Z., Polosukhin V., Wain J., Karimi-Shah B.A., Kim N.D. (2008). The lysophosphatidic acid receptor LPA1 links pulmonary fibrosis to lung injury by mediating fibroblast recruitment and vascular leak. Nat. Med..

[127] Tang N., Zhao Y., Feng R., Liu Y., Wang S., Wei W., Ding Q., An M.S., Wen J., Li L. (2014). Lysophosphatidic acid accelerates lung fibrosis by inducing differentiation of mesenchymal stem cells into myofibroblasts. J. Cell Mol. Med..

[128] Magkrioti C., Galaris A., Kanellopoulou P., Stylianaki E.-A., Kaffe E., Aidinis V. (2019). Autotaxin and chronic inflammatory diseases. J. Autoimmun..

[129] Funke M., Knudsen L., Lagares D., Ebener S., Probst C.K., Fontaine B.A., Franklin A., Kellner M., Kühnel M., Matthieu S. (2016). Lysophosphatidic acid signaling through the lysophosphatidic acid-1 receptor is required for alveolarization. Am. J. Respir. Cell Mol. Biol..

[130] Taleb S.J., Wei J., Mialki R.K., Dong S., Li Y., Zhao J., Zhao Y. (2021). A blocking peptide stabilizes lysophosphatidic acid receptor 1 and promotes lysophosphatidic acid-induced cellular responses. J. Cell. Biochem..

[131] Charbeneau R.P., Peters-Golden M. (2005). Eicosanoids: Mediators and therapeutic targets in fibrotic lung disease. Clin. Sci..

[132] Zannikou M., Barbayianni I., Fanidis D., Grigorakaki T., Vlachopoulou E., Konstantopoulos D., Fousteri M., Nikitopoulou I., Kotanidou A., Kaffe E. (2021). MAP3K8 regulates Cox-2-mediated prostaglandin E2 production in the lung and suppresses pulmonary inflammation and fibrosis. J. Immunol..

[133] Evans I.C., Barnes J.L., Garner I.M., Pearce D.R., Maher T.M., Shiwen X., Renzoni E.A., Wells A.U., Denton C.P., Laurent G.J. (2016). Epigenetic regulation of cyclooxygenase-2 by methylation of C8orf4 in pulmonary fibrosis. Clin. Sci..

[134] Hirata H., Arima M., Fukushima Y., Sugiyama K., Tokuhisa T., Fukuda T. (2013). Leukotriene C4 aggravates bleomycin-induced pulmonary fibrosis in mice. Respirology.

[135] Peters-Golden M., Bailie M., Marshall T., Wilke C., Phan S.H., Toews G.B., Moore B.B. (2002). Protection from pulmonary fibrosis in leukotriene-deficient mice. Am. J. Respir. Crit. Care Med..

[136] Shimbori C., Shiota N., Okunishi H. (2011). Effects of montelukast, a cysteinyl-leukotriene type 1 receptor antagonist, on the pathogenesis of bleomycin-induced pulmonary fibrosis in mice. Eur. J. Pharmacol..

[137] Izumo T., Kondo M., Nagai A. (2009). Effects of a leukotriene B4 receptor antagonist on bleomycin-induced pulmonary fibrosis. Eur. Respir. J..

[138] Hur J., Kang J.Y., Rhee C.K., Kim Y.K., Lee S.Y. (2018). The leukotriene receptor antagonist pranlukast attenuates airway remodeling by suppressing TGF-β signaling. Pulm. Pharmacol. Ther..

[139] Kida T., Ayabe S., Omori K., Nakamura T., Maehara T., Aritake K., Urade Y., Murata T. (2016). Prostaglandin D2 attenuates bleomycin-induced lung inflammation and pulmonary fibrosis. PLoS ONE.

[140] Dackor R.T., Cheng J., Voltz J.W., Card J.W., Ferguson C.D., Garrett R.C., Bradbury J.A., DeGraff L.M., Lih F.B., Tomer K.B. (2011). Prostaglandin E₂ protects murine lungs from bleomycin-induced pulmonary fibrosis and lung dysfunction. Am. J. Physiol. Lung Cell Mol. Physiol..

[141] Lovgren A.K., Jania L.A., Hartney J.M., Parsons K.K., Audoly L.P., Fitzgerald G.A., Tilley S.L., Koller B.H. (2006). COX-2-derived prostacyclin protects against bleomycin-induced pulmonary fibrosis. Am. J. Physiol. Lung Cell Mol. Physiol..

[142] Kountz T.S., Jairaman A., Kountz C.D., Stauderman K.A., Schleimer R.P., Prakriya M. (2021). Differential regulation of ATP- and UTP-evoked prostaglandin E2 and IL-6 production from human airway epithelial cells. J. Immunol..

[143] Weinberg E., Zeldich E., Weinreb M.M., Moses O., Nemcovsky C., Weinreb M. (2009). Prostaglandin E2 inhibits the proliferation of human gingival fibroblasts via the EP2 Receptor and Epac. J. Cell Biochem..

[144] Huang S., Wettlaufer S.H., Hogaboam C., Aronoff D.M., Peters-Golden M. (2007). Prostaglandin E(2) inhibits collagen expression and proliferation in patient-derived normal lung fibroblasts via E Prostanoid 2 receptor and CAMP signaling. Am. J. Physiol. Lung Cell Mol. Physiol..

[145] Kolodsick J.E., Peters-Golden M., Larios J., Toews G.B., Thannickal V.J., Moore B.B. (2003). Prostaglandin E2 inhibits fibroblast to myofibroblast transition via E. prostanoid receptor 2 signaling and cyclic adenosine monophosphate elevation. Am. J. Respir. Cell Mol. Biol..

[146] Mukherjee S., Sheng W., Michkov A., Sriarm K., Sun R., Dvorkin-Gheva A., Insel P.A., Janssen L.J. (2019). Prostaglandin E2 inhibits profibrotic function of human pulmonary fibroblasts by disrupting Ca^2+^ signaling. Am. J. Physiol. Lung Cell Mol. Physiol..

[147] Epa A.P., Thatcher T.H., Pollock S.J., Wahl L.A., Lyda E., Kottmann R.M., Phipps R.P., Sime P.J. (2015). Normal human lung epithelial cells inhibit transforming growth factor-β induced myofibroblast differentiation via prostaglandin E2. PLoS ONE.

[148] Lacy S.H., Epa A.P., Pollock S.J., Woeller C.F., Thatcher T.H., Phipps R.P., Sime P.J. (2018). Activated human T lymphocytes inhibit TGFβ-induced fibroblast to myofibroblast differentiation via prostaglandins D2 and E2. Am. J. Physiol. Lung Cell Mol. Physiol..

[149] Huang S.K., Fisher A.S., Scruggs A.M., White E.S., Hogaboam C.M., Richardson B.C., Peters-Golden M. (2010). Hypermethylation of PTGER2 confers prostaglandin E2 resistance in fibrotic fibroblasts from humans and mice. Am. J. Pathol..

[150] Bauman K.A., Wettlaufer S.H., Okunishi K., Vannella K.M., Stoolman J.S., Huang S.K., Courey A.J., White E.S., Hogaboam C.M., Simon R.H. (2010). The antifibrotic effects of plasminogen activation occur via prostaglandin E2 synthesis in humans and mice. J. Clin. Investig..

[151] Okunishi K., Sisson T.H., Huang S.K., Hogaboam C.M., Simon R.H., Peters-Golden M. (2011). Plasmin overcomes resistance to prostaglandin E2 in fibrotic lung fibroblasts by reorganizing protein kinase a signaling. J. Biol. Chem..

[152] Cartier A., Hla T. (2019). Sphingosine 1-phosphate: Lipid signaling in pathology and therapy. Science.

[153] Petrache I., Berdyshev E.V. (2016). Ceramide signaling and metabolism in pathophysiological states of the lung. Annu. Rev. Physiol..

[154] Schwalm S., Pfeilschifter J., Huwiler A. (2013). Sphingosine-1-phosphate: A janus-faced mediator of fibrotic diseases. Biochim. Biophys. Acta.

[155] Milara J., Navarro R., Juan G., Peiró T., Serrano A., Ramón M., Morcillo E., Cortijo J. (2012). Sphingosine-1-phosphate is increased in patients with idiopathic pulmonary fibrosis and mediates epithelial to mesenchymal transition. Thorax.

[156] Dhami R., He X., Schuchman E.H. (2010). Acid sphingomyelinase deficiency attenuates bleomycin-induced lung inflammation and fibrosis in mice. Cell Physiol. Biochem..

[157] Tatler A.L., Jenkins G. (2015). Sphingosine-1-phosphate metabolism: Can its enigmatic lyase promote the autophagy of fibrosis?. Thorax.

[158] Gong L., Wu X., Li X., Ni X., Gu W., Wang X., Ji H., Hu L., Zhu L. (2020). S1PR3 deficiency alleviates radiation-induced pulmonary fibrosis through the regulation of epithelial-mesenchymal transition by targeting MiR-495-3p. J. Cell Physiol..

[159] Huang L.S., Sudhadevi T., Fu P., Punathil-Kannan P.-K., Ebenezer D.L., Ramchandran R., Putherickal V., Cheresh P., Zhou G., Ha A.W. (2020). Sphingosine kinase 1/S1P signaling contributes to pulmonary fibrosis by activating Hippo/YAP pathway and mitochondrial reactive oxygen species in lung fibroblasts. Int. J. Mol. Sci..

[160] Knipe R.S., Spinney J.J., Abe E.A., Probst C.K., Franklin A., Logue A., Giacona F., Drummond M., Griffith J., Brazee P.L. (2021). Endothelial-specific loss of sphingosine-1-phosphate receptor 1 increases vascular permeability and exacerbates bleomycin-induced pulmonary fibrosis. Am. J. Respir. Cell Mol. Biol..

[161] Park S.-J., Im D.-S. (2019). Deficiency of sphingosine-1-phosphate receptor 2 (S1P2) attenuates bleomycin-induced pulmonary fibrosis. Biomol. Ther..

[162] Zhao J., Okamoto Y., Asano Y., Ishimaru K., Aki S., Yoshioka K., Takuwa N., Wada T., Inagaki Y., Takahashi C. (2018). Sphingosine-1-phosphate receptor-2 facilitates pulmonary fibrosis through potentiating IL-13 pathway in macrophages. PLoS ONE.

[163] Van Dyken S.J., Locksley R.M. (2013). Interleukin-4- and Interleukin-13-mediated alternatively activated macrophages: Roles in homeostasis and disease. Annu. Rev. Immunol..

[164] Martin-Medina A., Lehmann M., Burgy O., Hermann S., Baarsma H.A., Wagner D.E., De Santis M.M., Ciolek F., Hofer T.P., Frankenberger M. (2018). Increased extracellular vesicles mediate WNT5A signaling in idiopathic pulmonary fibrosis. Am. J. Respir. Crit. Care Med..

[165] Shaba E., Landi C., Carleo A., Vantaggiato L., Paccagnini E., Gentile M., Bianchi L., Lupetti P., Bargagli E., Prasse A. (2021). Proteome characterization of BALF extracellular vesicles in idiopathic pulmonary fibrosis: Unveiling undercover molecular pathways. Int J. Mol. Sci..

[166] Njock M.-S., Guiot J., Henket M.A., Nivelles O., Thiry M., Dequiedt F., Corhay J.-L., Louis R.E., Struman I. (2019). Sputum Exosomes: Promising biomarkers for idiopathic pulmonary fibrosis. Thorax.

[167] Parimon T., Yao C., Habiel D.M., Ge L., Bora S.A., Brauer R., Evans C.M., Xie T., Alonso-Valenteen F., Medina-Kauwe L.K. (2019). Syndecan-1 promotes lung fibrosis by regulating epithelial reprogramming through extracellular vesicles. JCI Insight.

[168] Kaur G., Maremanda K.P., Campos M., Chand H.S., Li F., Hirani N., Haseeb M.A., Li D., Rahman I. (2021). Distinct Exosomal MiRNA Profiles from BALF and lung tissue of COPD and IPF patients. Int. J. Mol. Sci..

[169] d’Alessandro M., Soccio P., Bergantini L., Cameli P., Scioscia G., Foschino Barbaro M.P., Lacedonia D., Bargagli E. (2021). Extracellular vesicle surface signatures in IPF patients: A multiplex bead-based flow cytometry approach. Cells.

[170] Guiot J., Cambier M., Boeckx A., Henket M., Nivelles O., Gester F., Louis E., Malaise M., Dequiedt F., Louis R. (2020). macrophage-derived exosomes attenuate fibrosis in airway epithelial cells through delivery of antifibrotic MiR-142-3p. Thorax.

[171] Kadota T., Fujita Y., Araya J., Watanabe N., Fujimoto S., Kawamoto H., Minagawa S., Hara H., Ohtsuka T., Yamamoto Y. (2021). Human bronchial epithelial cell-derived extracellular vesicle therapy for pulmonary fibrosis via inhibition of TGF-β-WNT crosstalk. J. Extracell. Vesicles.

[172] Hough K.P., Wilson L.S., Trevor J.L., Strenkowski J.G., Maina N., Kim Y.-I., Spell M.L., Wang Y., Chanda D., Dager J.R. (2018). Unique lipid signatures of extracellular vesicles from the airways of asthmatics. Sci. Rep..

[173] Torregrosa Paredes P., Esser J., Admyre C., Nord M., Rahman Q.K., Lukic A., Rådmark O., Grönneberg R., Grunewald J., Eklund A. (2012). Bronchoalveolar lavage fluid exosomes contribute to cytokine and leukotriene production in allergic asthma. Allergy.

[174] Sato S., Chong S.G., Upagupta C., Yanagihara T., Saito T., Shimbori C., Bellaye P.-S., Nishioka Y., Kolb M.R. (2021). Fibrotic extracellular matrix induces release of extracellular vesicles with pro-fibrotic MiRNA from fibrocytes. Thorax.

[175] Lacy S.H., Woeller C.F., Thatcher T.H., Pollock S.J., Small E.M., Sime P.J., Phipps R.P. (2019). Activated Human Lung Fibroblasts Produce Extracellular Vesicles with Antifibrotic Prostaglandins. Am. J. Respir. Cell Mol. Biol..

[176] Mayr C.H., Simon L.M., Leuschner G., Ansari M., Schniering J., Geyer P.E., Angelidis I., Strunz M., Singh P., Kneidinger N. (2021). Integrative analysis of cell state changes in lung fibrosis with peripheral protein biomarkers. EMBO Mol. Med..

[177] Barochia A.V., Kaler M., Weir N., Gordon E.M., Figueroa D.M., Yao X., Lemma WoldeHanna M., Sampson M., Remaley A.T., Grant G. (2021). Serum levels of small HDL particles are negatively correlated with death or lung transplantation in an observational study of idiopathic pulmonary fibrosis. Eur. Respir. J..

[178] Podolanczuk A.J., Raghu G., Tsai M.Y., Kawut S.M., Peterson E., Sonti R., Rabinowitz D., Johnson C., Barr R.G., Hinckley Stukovsky K. (2017). Cholesterol, lipoproteins and subclinical interstitial lung disease: The MESA study. Thorax.

[179] Mallampalli R.K., Ryan A.J., Carroll J.L., Osborne T.F., Thomas C.P. (2002). Lipid deprivation increases surfactant phosphatidylcholine synthesis via a sterol-sensitive regulatory element within the CTP: Phosphocholine cytidylyltransferase promoter. Biochem. J..

[180] Ryan A.J., Medh J.D., McCoy D.M., Salome R.G., Mallampalli R.K. (2002). Maternal loading with very low-density lipoproteins stimulates fetal surfactant synthesis. Am. J. Physiol. Lung Cell Mol. Physiol..

[181] Yan F., Wen Z., Wang R., Luo W., Du Y., Wang W., Chen X. (2017). Identification of the lipid biomarkers from plasma in idiopathic pulmonary fibrosis by lipidomics. BMC Pulm. Med..

[182] Nambiar S., Clynick B., How B.S., King A., Walters E.H., Goh N.S., Corte T.J., Trengove R., Tan D., Moodley Y. (2021). There is detectable variation in the lipidomic profile between stable and progressive patients with idiopathic pulmonary fibrosis (IPF). Respir. Res..

[183] Rangarajan S., Bone N.B., Zmijewska A.A., Jiang S., Park D.W., Bernard K., Locy M.L., Ravi S., Deshane J., Mannon R.B. (2018). Metformin reverses established lung fibrosis in a bleomycin model. Nat. Med..

[184] Zhang X., Cai Y., Zhang W., Chen X. (2018). Quercetin ameliorates pulmonary fibrosis by inhibiting SphK1/S1P Signaling. Biochem. Cell Biol..

[185] Zhu B., Ma A.-Q., Yang L., Dang X.-M. (2013). Atorvastatin attenuates bleomycin-induced pulmonary fibrosis via suppressing INOS expression and the CTGF (CCN2)/ERK signaling pathway. Int. J. Mol. Sci..

[186] Mammoliti O., Palisse A., Joannesse C., El Bkassiny S., Allart B., Jaunet A., Menet C., Coornaert B., Sonck K., Duys I. (2021). Discovery of the S1P2 antagonist GLPG2938 (1-[2-Ethoxy-6-(Trifluoromethyl)-4-Pyridyl]-3-[[5-Methyl-6-[1-Methyl-3-(Trifluoromethyl)Pyrazol-4-Yl]Pyridazin-3-Yl]Methyl]Urea), a preclinical candidate for the treatment of idiopathic pulmonary fibrosis. J. Med. Chem..

[187] Kreuter M., Bonella F., Maher T.M., Costabel U., Spagnolo P., Weycker D., Kirchgaessler K.-U., Kolb M. (2017). Effect of statins on disease-related outcomes in patients with idiopathic pulmonary fibrosis. Thorax.

[188] M Lambert E., A Wuyts W., Yserbyt J., De Sadeleer L.J. (2021). Statins: Cause of fibrosis or the opposite? Effect of cardiovascular drugs in idiopathic pulmonary fibrosis. Respir. Med..

[189] Chang M., Xu G., Xiong C., Yang X., Yan S., Tao Y., Li H., Li Y., Yao S., Zhao Y. (2021). Alpha-lipoic acid attenuates silica-induced pulmonary fibrosis by improving mitochondrial function via AMPK/PGC1α pathway activation in C57BL/6J Mice. Toxicol. Lett..

[190] Li H., Hao Y., Zhang H., Ying W., Li D., Ge Y., Ying B., Cheng B., Lian Q., Jin S. (2017). Posttreatment with Protectin DX ameliorates bleomycin-induced pulmonary fibrosis and lung dysfunction in mice. Sci. Rep..

[191] Sun Q., Wu Y., Zhao F., Wang J. (2017). Maresin 1 inhibits transforming growth factor-Β1-induced proliferation, migration and differentiation in human lung fibroblasts. Mol. Med. Rep..

[192] Zhao H., Chan-Li Y., Collins S.L., Zhang Y., Hallowell R.W., Mitzner W., Horton M.R. (2014). Pulmonary delivery of docosahexaenoic acid mitigates bleomycin-induced pulmonary fibrosis. BMC Pulm. Med..

[193] Li X., Xie M., Lu C., Mao J., Cao Y., Yang Y., Wei Y., Liu X., Cao S., Song Y. (2020). Design and synthesis of leukotriene A4 hydrolase inhibitors to alleviate idiopathic pulmonary fibrosis and acute lung injury. Eur. J. Med. Chem..

[194] Ji Y.-D., Luo Z.-L., Chen C.-X., Li B., Gong J., Wang Y.-X., Chen L., Yao S.-L., Shang Y. (2018). BML-111 suppresses TGF-Β1-Induced lung fibroblast activation in vitro and decreases experimental pulmonary fibrosis in vivo. Int. J. Mol. Med..

[195] Ninou I., Kaffe E., Müller S., Budd D.C., Stevenson C.S., Ullmer C., Aidinis V. (2018). Pharmacologic targeting of the ATX/LPA axis attenuates bleomycin-induced pulmonary fibrosis. Pulm. Pharmacol. Ther..

[196] Matralis A.N., Afantitis A., Aidinis V. (2019). Development and therapeutic potential of autotaxin small molecule inhibitors: From bench to advanced clinical trials. Med. Res. Rev..

[197] Pascual G., Avgustinova A., Mejetta S., Martín M., Castellanos A., Attolini C.S.-O., Berenguer A., Prats N., Toll A., Hueto J.A. (2017). Targeting metastasis-initiating cells through the fatty acid receptor CD36. Nature.

[198] Nath A., Chan C. (2016). Genetic alterations in fatty acid transport and metabolism genes are associated with metastatic progression and poor prognosis of human cancers. Sci. Rep..

[199] Wang J., Li Y. (2019). CD36 tango in cancer: Signaling pathways and functions. Theranostics.

[200] Wang X., Chen Y., Lv L., Chen J. (2009). Silencing CD36 gene expression results in the inhibition of latent-TGF-Beta1 activation and suppression of silica-induced lung fibrosis in the rat. Respir. Res..

[201] Trappe A., Donnelly S.C., McNally P., Coppinger J.A. (2021). Role of extracellular vesicles in chronic lung disease. Thorax.

[202] Yamada M. (2021). Extracellular vesicles: Their emerging roles in the pathogenesis of respiratory diseases. Respir. Investig..

[203] Abreu S.C., Lopes-Pacheco M., Weiss D.J., Rocco P.R.M. (2021). Mesenchymal stromal cell-derived extracellular vesicles in lung diseases: Current status and perspectives. Front. Cell Dev. Biol..

[204] Massa M., Croce S., Campanelli R., Abbà C., Lenta E., Valsecchi C., Avanzini M.A. (2020). Clinical Applications of Mesenchymal Stem/Stromal Cell Derived Extracellular Vesicles: Therapeutic Potential of an Acellular Product. Diagnostics.

[205] Dinh P.C., Paudel D., Brochu H., Popowski K.D., Gracieux M.C., Cores J., Huang K., Hensley M.T., Harrell E., Vandergriff A.C. (2020). Inhalation of lung spheroid cell secretome and exosomes promotes lung repair in pulmonary fibrosis. Nat Commun..

[206] Tan J.L., Lau S.N., Leaw B., Nguyen H.P.T., Salamonsen L.A., Saad M.I., Chan S.T., Zhu D., Krause M., Kim C. (2018). Amnion epithelial cell-derived exosomes restrict lung injury and enhance endogenous lung repair. Stem Cells Transl. Med..

[207] Palmer S.M., Snyder L., Todd J.L., Soule B., Christian R., Anstrom K., Luo Y., Gagnon R., Rosen G. (2018). Randomized, double-blind, placebo-controlled, phase 2 trial of BMS-986020, a lysophosphatidic acid receptor antagonist for the treatment of idiopathic pulmonary fibrosis. Chest.

[208] Khalil N., Manganas H., Ryerson C.J., Shapera S., Cantin A.M., Hernandez P., Turcotte E.E., Parker J.M., Moran J.E., Albert G.R. (2019). Phase 2 clinical trial of PBI-4050 in patients with idiopathic pulmonary fibrosis. Eur. Respir. J..

[209] Nguyen Q.T., Nsaibia M.J., Sirois M.G., Calderone A., Tardif J.-C., Fen Shi Y., Ruiz M., Daneault C., Gagnon L., Grouix B. (2020). PBI-4050 reduces pulmonary hypertension, lung fibrosis, and right ventricular dysfunction in heart failure. Cardiovasc. Res..

